# A dynamic allosteric pathway underlies Rad50 ABC ATPase function in DNA repair

**DOI:** 10.1038/s41598-018-19908-8

**Published:** 2018-01-26

**Authors:** Zachary K. Boswell, Samiur Rahman, Marella D. Canny, Michael P. Latham

**Affiliations:** Department of Chemistry and Biochemistry, Texas Tech University, Lubbock, TX 79423 USA

## Abstract

The Mre11-Rad50 protein complex is an initial responder to sites of DNA double strand breaks. Many studies have shown that ATP binding to Rad50 causes global changes to the Mre11-Rad50 structure, which are important for DNA repair functions. Here we used methyl-based NMR spectroscopy on a series of mutants to describe a dynamic allosteric pathway within Rad50. Mutations result in changes in the side chain methyl group chemical environment that are correlated with altered nanosecond timescale dynamics. We also observe striking relationships between the magnitude of chemical shift perturbations and Rad50 and Mre11 activities. Together, these data suggest an equilibrium between a ground state and an “active” dimerization competent state of Rad50 that has locally altered structure and dynamics and is poised for ATP-induced dimerization and eventual ATP hydrolysis. Thus, this sparsely populated intermediate is critical for Mre11-Rad50-directed DNA double strand break repair.

## Introduction

**Figure 1:**
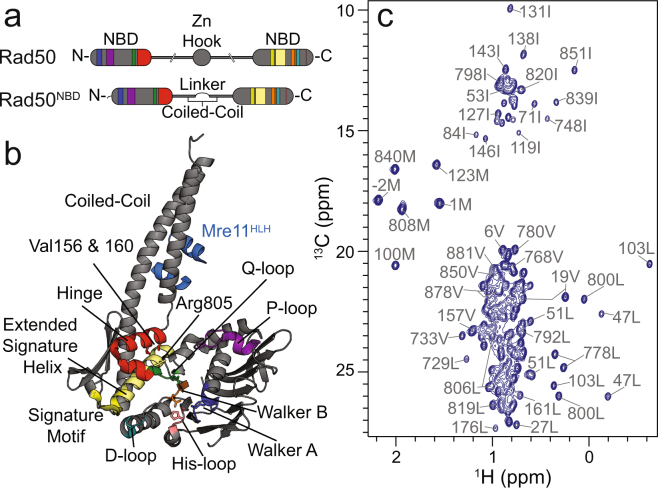
Domain architecture and NMR spectra of Rad50. (**a**) Cartoon representation of full length Rad50 (top) and Rad50^NBD^ (bottom). (**b**) Crystal structure of *P. furiosus* Mre11^HLH^-Rad50^NBD^ (3QKS)^18^. Colors in (**a**) and (**b**) illustrate conserved domains important for Rad50 function. (**c**) 2D ^13^C,^1^H methyl-TROSY HMQC^25^ spectrum of Mre11^HLH^-Rad50^NBD^ collected at 14.1 T and 50 °C. The side chain methyl group assignments are given.

The conserved and essential Mre11-Rad50 complex in archaea and bacteria and larger Mre11-Rad50-Nbs1 (or Xrs1) complex in higher eukaryotes is responsible for detecting and initiating the repair of DNA double strand breaks (DSBs)^1,2^. Mre11 is an obligate dimer that has Mn^2+^-dependent 3′-to-5′ exonuclease and ssDNA endonuclease activity^3,4^. Each Mre11 dimer is bound to two molecules of Rad50 (i.e., M_2_R_2_), a member of the ATP binding cassette (ABC) family of proteins^5,6^ whose structure is shown in Fig. 1. Nbs1 (or Xrs1) is a flexible scaffolding protein that uses phospho-protein binding domains to recruit downstream effectors to the site of the DNA break^7,8^. The proper function of this protein complex is absolutely required for the repair of mutagenic or cytotoxic DNA DSBs^1,2^. A number of hypomorphic mutations have been observed in Mre11-Rad50-Nbs1 that lead to neuromuscular developmental disorders, immunodeficiency, and a predisposition to cancer^9–11^, and spontaneous mutations have been observed in a number of cancer types characterized by gross genomic instabilities^12,13^.

Rad50 has a unique structural arrangement whereby the N- and C-terminal sub-domains, which are separated by ~200–600 Å coiled-coil and apical Zn-hook domains, fold back onto each other to form a functional ABC ATPase domain (“NBD”s in Fig. 1a)^5,6^. As with other members of this protein family, the binding of two ATP molecules induces dimerization of the two Rad50 molecules within the M_2_R_2_ complex^6^. Several high-resolution X-ray crystal structures have revealed that Rad50 dimerization results in large structural rearrangements of the entire M_2_R_2_ complex, dramatically rotating Rad50 from an “open” to a “closed” form when ATP is bound^14–18^. It is known that the functionality of the M_2_R_2_ complex changes depending on the ATP state of Rad50: Mre11 is exonuclease active in the ATP-free, “open” form; M_2_R_2_ DNA tethering activities appear to be stimulated in the ATP-bound, “closed” form; and Mre11 ssDNA endonuclease activity may arise from a proposed ADP-bound intermediate state^4,19–21^. X-ray crystal structures and biochemical data have shown that ATP binding leads to the local rearrangement of salt-bridge and hydrogen bonding interactions within Rad50^18,19^. This shuffling of ~20 charge-pair interactions is thought to transmit Rad50 ATP binding state information through the coiled-coil domain to Mre11 and out to the apical Zn-hook domain - long-range allostery that would thus span several hundreds of Ångstroms^18,22,23^. Included in these ionic interactions is a critical contact involving an arginine residue in the extended signature helix, the so called basic switch (R805 in Fig. 1b). Disruption of this basic switch via mutation of this residue from arginine to glutamate leads to changes in Rad50 ATP hydrolysis activity and ATP-dependent dimerization and affects the ability of Mre11-Rad50-Nbs1 to repair DNA DSBs, an indication that long-range allostery is being altered^19^.

Here, we used nuclear magnetic resonance (NMR) spectroscopy to describe a system of residues spanning the Rad50 ABC ATPase domain whose chemical shifts change upon mutation to the hinge region (V156 and V160) and basic switch (R805) (see Fig. 1b). Analysis of these chemical shift changes via a covariation method reveals three distinct clusters within Rad50. These clusters form an allosteric network that couples residues in the vicinity of ATP binding to residues at the base of the coiled-coil and Mre11 interacting domains as well as those in the Rad50 dimerization regions. Methyl group relaxation data shows that this pathway is not static in nature, as the amplitudes of methyl group psec – nsec timescale motions correlate with the chemical shift changes. Furthermore, we show that interruption of this network leads to changes in Rad50 that increase both its propensity for dimerization and ATP hydrolysis activity. We finally demonstrate that disruption of this allosteric pathway within Rad50 has a profound effect on Mre11 exonuclease activity. When the effects of mutation on the structure and dynamics are compared with biochemical activity, we see significant correlations for methyl groups in two of the three clusters. Together, these data suggest a model whereby many local changes within the Rad50^NBD^ structure are coupled with a general increase in side chain flexibility, ATP-induced dimerization, and ATP hydrolysis – activities required for M_2_R_2_-directed DNA DSB repair activities.

## Results

### Side chain methyl group assignment of *P. furiosus* Rad50^NBD^

*P. furiosus* (Pf) Rad50 and Mre11 have been used as a model M_2_R_2_ complex for a number of structural biology and biophysical studies^4,5,17–19^. Our initial NMR studies have focused on a common truncated form of Pf Rad50 containing the N- and C-terminal ABC-ATPase nucleotide binding sub-domains connected by a six-residue flexible linker (hereafter, Rad50^NBD^) as depicted in Fig. 1a. Because of the size of the Mre11_2_-Rad50^NBD^_2_ complex (~160 kDa), we utilized uniformly deuterated, side chain methyl Ileδ1-^13^CH_3_; Leuδ/Valγ-^13^CH_3_/^12^CD_3_; Metε-^13^CH_3_-labeled (referred to as ILVM-labeled) protein samples coupled with methyl-Transverse Relaxation Optimized SpectroscopY (TROSY)^24,25^. For ILVM side chain methyl group assignments of Rad50, we made monomeric ILVM-labeled Rad50^NBD^ in complex with protonated Mre11 helix-loop-helix (Mre11 ^HLH^; light blue in Fig. 1b) domain. Mre11^HLH^ is responsible for complex formation between Rad50 and full length Mre11^18^ and is necessary for well-behaved Rad50^NBD^ (see Supplementary Information). Figure 1c shows the 2D ^13^C,^1^H methyl-TROSY correlation spectrum of Mre11^HLH^ - Rad50^NBD^; 152 out of 168 (~90%) Rad50^NBD^ side chain methyl group assignments have been determined through the comparison of experimental methyl-methyl distance observations (nuclear Overhauser effects – NOEs) with theoretical distances calculated from a Pf Rad50^NBD^ crystal structure (pdb: 1II8^5^). Methyl group assignments were supplemented and validated with conservative mutations of ILVM-residues based on a sequence alignment^26^ or with the mutation of surface ILVM-residues to cysteine followed by covalent modification with a paramagnetic spin label^27^. The spin label broadens resonances in the methyl-TROSY correlation spectra corresponding to methyl groups within a ~20 Å radius of the probe^28^ (Supplementary Fig. S1), confirming which residues are in its vicinity.

### Mutations in the Rad50^NBD^ hinge region and basic switch reveal an allosteric network coupling ATP binding to the base of the coiled-coil domain

**Figure 2:**
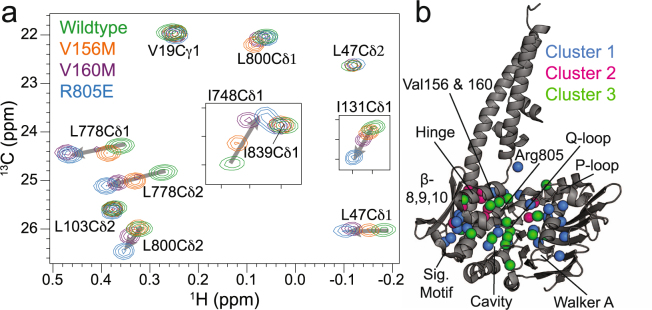
Mutations reveal an allosteric network in Rad50^NBD^. (**a**) 2D ^13^C,^1^H methyl-TROSY HMQC spectral overlay of WT and mutant Mre11^HLH^-Rad50^NBD^ recorded at 14.1 T and 50 °C. Arrows highlight side chain methyl groups experiencing CSPs. (**b**) Structure of Mre11^HLH^-Rad50^NBD^ emphasizing side chain methyl groups whose CSPs upon mutation cluster according to CHESCA as described in the Methods. Clusters are listed in Supplementary Table S1 and were determined via the dendrogram in Supplementary Fig. S4. Methyl groups not affected by the mutations are not shown.

The hinge region of Rad50 (also called the signature-coupling helices) is adjacent to the conserved ABC-ATPase signature motif (Fig. 1b) and couples ATP binding, as sensed by the Q-loop and Walker B motif, to the base of the coiled-coil domain, where Mre11^HLH^ binds, through a series of switchable ionic and hydrogen bonding interactions^18,19^. This region posed a particularly difficult area in the structure for the NOE-based assignment process because it is densely packed with ILVM residues, and an unambiguous network of side chain methyl groups was challenging to identify from methyl-methyl NOE data. Four mutations were therefore made to this region to aid in assignments: V156M, V157M, and V160M in the signature-coupling α2 helix and L163M at the base of the coiled-coil domain. Even though these mutations did indeed provide methyl group assignments via the disappearance of peaks for the mutated residue and changes to peak positions for nearby residues in the methyl-TROSY correlation spectra (Supplementary Fig. S2), other widespread and systematic changes were surprisingly observed in the data for V156M, V157M, and V160M, shown in the spectral overlay of Fig. 2a (L163M only showed the expected local changes). These chemical shift perturbations (CSPs) radiated out from the site of mutation, and in the case of V156M and V160M, included regions where ATP binds, the base of the coiled-coil domain, the P-loop, and β-strands 8, 9, and 10, which are adjacent to the signature motif and would be near the ATP-binding site of the second Rad50^NBD^ upon dimerization (Figs 1b and 2b): a network that spans ~50 Å. Also shown in the overlay of methyl-TROSY correlation spectra in Fig. 2a is the data for the Mre11^HLH^-Rad50^NBD^(R805E) basic switch mutant (full spectra presented in Supplementary Fig. S2). Again, a number of expected CSPs were observed for residues in the area of the structure adjacent to the R805E mutation, and widespread methyl group CSPs were also observed in the same regions that were affected by V156M and V160M.

We initially used a median-absolute-deviations approach^29,30^, a common method to find outliers within a distribution, to identify side chain methyl groups that have substantial changes in their resonance positions upon mutation (Supplementary Fig. S3). For the residues that have significant deviations (modified *Z-score* > 0.25), analysis of the CSPs like those shown in Fig. 2a revealed a linear change in peak position as a function of mutation. The linear movement demonstrates that these CSPs are not simply the result of structural changes resulting from the mutations to methionine, as one would expect those effects to not have systematic differences in the methyl-TROSY spectra. Instead, the data of Fig. 2a can most easily be explained as a shift in equilibrium between two states, where each of the mutants has a different population of the two states, that is fast on the NMR chemical shift timescale and results in the observed population weighted average of chemical shifts^31^. We observed significant CSPs in the network of salt-bridge and hydrogen bond interactions that connect the Q-loop and R805 to the P-loop (Supplementary Fig. S3)^18^. The disruption of this network has been noted in the crystal structure of a further truncated Pf Rad50^NBD^(R805E) construct (i.e., it does not contain any of the coiled-coil domain) and is thought to be responsible for the increased ATP-binding affinity observed for the R805E mutation^19^ (see below). Furthermore, we also observed methyl CSPs at the base of the coiled-coil domain and in methyl groups down the extended signature helix to the R797 basic switch residue, which couples this helix to the ABC signature motif and is important for dimerization^18^. Perturbations to the extended signature helix also affected methyl groups that line a hydrophobic cavity that has been shown to collapse upon ATP binding^19^. However, a careful inspection of the data shows that the effect of mutation is not uniform for every residue. For example, the V160M and R805E mutations have the same CSP for L778, whereas the CSP for R805E is greater than V160M for L800 (Fig. 2a).

**Figure 3:**
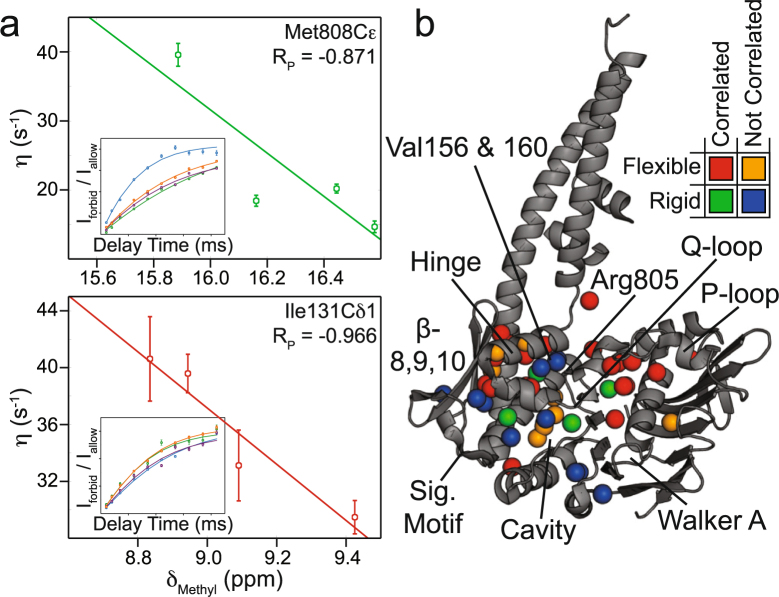
Chemical shift perturbations and dynamics changes are correlated. (**a**) Representative *η vs δ*_*Methyl*_ scatter plots for Met808Cε (*top*) and Ile131Cδ1 (*bottom*). *δ*_*Methyl*_ values were determined according to equation (1), see Supporting Information. Pearson’s correlation coefficients (R_P_) are given in the upper right corner. *Insets* show the build-up curves for the ratio of intensities arising from methyl group ^1^H triple-quantum “forbidden” experiments^36^ (I_forbid_/I_allow_) *vs*. relaxation delay time for wildtype and mutant Mre11^HLH^-ILVM labeled Rad50^NBD^. These data were fit to equation (3) to determine the *η* rates^36^, as described in the Methods, with errors determined from the covariance matrix of the fit^48^. The coloring of the curves corresponds to the spectra of the mutants in Fig. 2a. (**b**) Structure of Mre11^HLH^-Rad50^NBD^ showing side chain methyl groups with altered dynamics upon mutation. Red and orange spheres represent methyl groups that become more flexible upon mutation, whereas green and blue spheres represent methyl groups that become more rigid upon mutation. Red and green coloring denotes “Correlated” methyl groups with significant CSPs upon mutation (i.e., the range in *δ*_*Methyl*_ > 0.13 ppm) that also have a correlation for *η vs δ*_*Methyl*_ of |R_P_| > 0.7. Orange and blue coloring denotes “Not Correlated” methyl groups with small CSPs upon mutation (i.e., the range in *δ*_*Methyl*_ < 0.13 ppm) but have a large difference in *η* rates between wildtype and the mutants (|η_WT_ – the average η_mutants_| > 8 sec^−1^).

We therefore turned to chemical shift covariance analysis (CHESCA), which is a method to cluster CSPs in fast exchange like those in Fig. 2a into groups of residues experiencing the same shift in populations upon perturbation^31–34^. Using the median-absolute-deviations filtered methyl residues (Supplementary Fig. S3), CHESCA produced three distinct clusters of side chain methyl groups within Rad50^NBD^ (Supplementary Fig. S4 and Supplementary Table S1). Figure 2b highlights those clusters on the structure of Mre11^HLH^-Rad50^NBD^. Cluster 1 (blue spheres in Fig. 2b) largely encompasses the extended signature motif, β-strands 8, 9, and 10, P-loop, and Walker A motif; cluster 2 (magenta spheres in Fig. 2b) consists of residues near the sites of the mutations but also includes methyl groups along the salt-bridge and hydrogen bonding network that switch upon ATP binding^18^; cluster 3 (green spheres in Fig. 3b) contains methyl groups within the hydrophobic core – an area that experiences compaction upon dimerization^19^. Again, these three clusters connect the nucleotide binding site through the Q-loop, R805 basic switch, and signature-coupling helix α2 to the coiled-coil domain at one end of Rad50^NBD^ and to the base of the signature motif at the other.

### The Rad50^NBD^ allosteric network is a dynamic system

We next questioned the effect that the above mutations would have on methyl group dynamics. The data in Fig. 2a is indicative of fast conformational exchange on the chemical shift timescale, which can be quantified with relaxation dispersion Carr-Purcell-Meiboom-Gill (CPMG) methods^35^. However, we did not observe any relaxation dispersions in wildtype or mutant Mre11^HLH^-Rad50^NBD^ (see Supporting Information) indicating that if exchange is happening it is faster than the msec timescale. Fast timescale (psec – nsec) motions were quantified from the rate of the build-up for “forbidden” methyl ^1^H triple quantum coherences^36^ (*η*) in Mre11^HLH^- Rad50^NBD^ complexes (Fig. 3a, *insets*). The calculated *η* rates directly report on the amplitude of methyl side chain motion (order parameter - ${S}_{axis}^{2}$) as well as the global tumbling time (*τ*_*c*_)^36^. These experiments were performed on the nucleotide-free state for each mutation, where Mre11^HLH^-Rad50^NBD^ is monomeric, and therefore relate to changes in methyl group dynamics upon mutation and not changes in global tumbling resulting from dimerization. When the *η* rates for each Rad50^NBD^ (i.e., WT or V156M, V160M, or R805E) are plotted against the weighted combined methyl chemical shift^37^ (*δ*_*Methyl*_), as shown in Fig. 3a (and Supplementary Table 1), a clear linear relationship is observed for many of the residues that had significant chemical shift deviations upon mutation (i.e., the range in *δ*_*Methyl*_ > 0.13 ppm corresponding to a modified *Z-score* > 0.25). Thus, the amplitudes of methyl side chain dynamics also experience a population weighted shift upon mutation. We then analyzed the trend in the *η* rates versus *δ*_*Methyl*_ to extract a change in the amplitude of the methyl group dynamics upon mutation. The changes in fast timescale methyl dynamics are highlighted on the structure of Mre11^HLH^-Rad50^NBD^ in Fig. 3b: red and green spheres represent methyl groups with *η* rate *vs. δ*_*Methyl*_ correlations that are experiencing increasing and decreasing dynamics, respectively. We also examined methyl groups with small changes in methyl chemical shift (range in *δ*_*Methyl*_ < 0.13 ppm). Although the local environment of these methyl groups was not affected by mutations, their fast timescale dynamics might be. Indeed, the orange and blue spheres in Fig. 3b represent those methyl groups with small changes in *δ*_*Methyl*_ but with a substantial increase or decrease in side chain mobility, respectively. Together, these dynamics data, shown on the structure in Fig. 3b, illustrate a dynamic allosteric network that is generally becoming more flexible upon mutation. Specifically, we see increased flexibility (red and orange spheres) in and around the P-loop, the hinge (signature coupling helix α2) and base of the coiled-coil domain, including β-strands 8, 9, and 10, as well as parts of the extended signature helix near a hydrophobic cavity. On the other hand, isolated regions where the hinge (both signature coupling helices α1 and α2) interacts with the extended signature helix as well as the area around the H-loop are becoming more rigid (green and blue spheres).

### Changes in the Rad50^NBD^ allosteric network affect Rad50 activity

To determine the functional role of the allosteric network shown in Figs 2b and 3b, we performed biochemical activity assays on Rad50^NBD^ to assess changes in ATP and ADP affinity, ATP hydrolysis, and ATP-induced dimerization upon mutation. We hypothesized that since the mutations near the coiled-coil domain altered the structure and dynamics of methyl groups at the sites of ATP binding and Rad50 dimerization then these biochemical processes would be affected by mutation as well.

ATP binding affinities were determined by monitoring the increase in the fluorescence polarization of a BODIPY-ATP analog as a function of increasing Mre11^HLH^-Rad50^NBD^ concentration. From these titrations (Supplementary Figs S5 and S6), we determined a K_D,ATP_ of 16.9 ± 3.9 μM for the wildtype complex. As predicted from our hypothesis, mutations to the signature coupling helix α2 (V156M and V160M) and basic switch (R805E) did result in altered ATP binding affinities. The experimentally determined K_D,ATP_s (Supplementary Table 2) for the mutants show a lower K_D,ATP_ (i.e., a higher affinity) compared to wildtype. We used fluorescence resonance energy transfer (FRET) to determine the affinity of Rad50^NBD^ for the hydrolysis product ADP. Titration of Mre11^HLH^-Rad50^NBD^ to a modified MANT-ADP (Supplementary Figs S5 and S6) yielded an increase in FRET as the acceptor MANT-ADP comes into proximity of a Rad50^NBD^ tryptophan residue, likely W782^19^. From these data, a K_D,ADP_ of 2.3 ± 0.2 μM was calculated for wildtype Mre11^HLH^-Rad50^NBD^, and Supplementary Table 2 demonstrates that mutations to the hinge region and basic switch have similar K_D,ADP_ values as wildtype.

Next, Rad50^NBD^ ATP hydrolysis Michaelis-Menten kinetics were calculated from the increase in inorganic phosphate product concentration, as detected by the BioMol green reagent. Because of the very low rate of ATP hydrolysis observed for isolated Rad50^NBD^, we performed this assay with full-length Mre11_2_-Rad50^NBD^_2_ complex, which has a much greater rate of ATP hydrolysis^5^. Similar to ATP binding, we observed a ~2-fold lower K_M_ for V160M and R805E mutants as compared to WT (Supplementary Table 2 and Supplementary Fig. S5), while the k_cat_ for these mutants increased by nearly 4-fold. V156M has Michaelis-Menten parameters that fall between WT and V160M/R805E. Finally, the influence of the mutations on ATP-induced dimerization was assessed by size exclusion chromatography. As previously observed, the R805E mutation has a dramatic effect on Rad50^NBD^ ATP-induced dimerization, increasing the dimer population to ~79% compared to the ~35% seen for wildtype (Supplementary Table 2 and Supplementary Fig. S5)^19^. In line with the results above, the hinge mutations also serve to enhance Mre11^HLH^-Rad50^NBD^ ATP-induced dimerization, shifting the population of dimer upon ATP binding to 64% and 57% for V156M and V160M, respectively.

**Figure 4:**
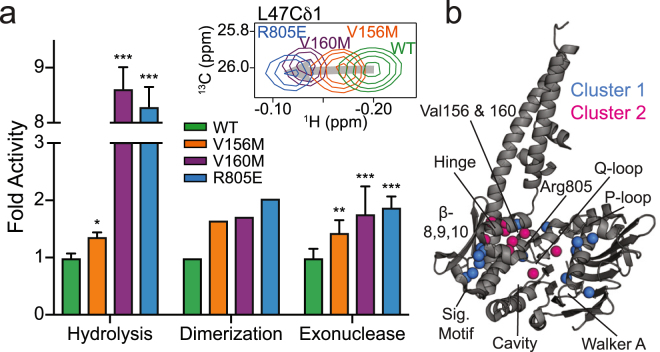
Basic switch and hinge region mutations affect Rad50 activity. (**a**) Bar chart showing the effect of mutation, relative to wildtype activity, for ATP hydrolysis (*left* - k_cat_/K_M_), ATP-dependent dimerization (*middle* - % dimerization), and Mre11 exonuclease activity (*right* – relative fluorescence). Green, orange, purple, and blue bars represent wildtype, V156M, V160M, and R805E, respectively. The order of the bars follows the general order of peaks that experience significant CSPs upon mutation. For ATP hydrolysis and exonuclease assays, bars represent the average of at least three independent measurements, and the error bars are the standard deviation of the replicate experiments. Dimerization assays were performed once. *^,^ ** and *** Represent p-values less than 0.05, 0.01, and 0.001, respectively. The *inset* above shows the CSPs for L47Cδ1. (**b**) Structure of Mre11^HLH^-Rad50^NBD^ highlighting the side chain methyl groups that have a |mean[R_P,Hydrolysis_, R_P,Dimerization_, R_P,Exonuclease_]| > 0.65.

Figure 4a illustrates the relative effect that each mutation has on Rad50^NBD^ ATP hydrolysis and ATP-induced dimerization activities. For each of these, the bars are ordered based on the general movement of peak positions away from WT resonances in the methyl-TROSY correlation spectra (gray arrows in Fig. 2a and in the upper inset of Fig. 4a). This representation shows the relationship between peak position in Fig. 2a and a higher rate of ATP hydrolysis (Fig. 4a, *left*) and a greater propensity to form ATP-induced dimer (Fig. 4a, *middle*). Importantly, this relationship demonstrates that altering the Rad50^NBD^ dynamic allosteric network highlighted in Figs 2b and 3b results in increased activities, which could possibly arise from either lowering the energy barrier for Rad50^NBD^ activity and/or increasing the population of an “active” Rad50^NBD^ conformation in solution (i.e., conformational capture).

### Mutations that probe Rad50^NBD^ allostery affect Mre11 exonuclease activity

Since a correlation was observed between the equilibrium peak positions of the hinge region and basic switch mutations in 2D ^13^C,^1^H methyl-TROSY spectra and Rad50^NBD^ activity, we next sought to determine if these Rad50 mutations had any effect on Mre11 nuclease activity. 3′-to-5′ Mn^2+^-dependent Mre11 exonuclease activity was monitored as an increase in fluorescence of a 2-aminopurine (2-AP) nucleotide analog, which has low fluorescence when incorporated into a DNA duplex but high fluorescence as the free nucleotide. This probe is placed at the second position from the 3′-end of a 29 bp DNA duplex and is released upon exonuclease cleavage of the DNA. As expected, the presence of the non-hydrolyzable ATP analog AMP-PNP resulted in a dramatic reduction in exonuclease activity, as the closed form of the M_2_R_2_ complex is stabilized and the exonuclease active site of Mre11 is occluded (Supplementary Fig. S7)^17,38,39^. Although the Rad50 mutations did not have a significant effect on Mre11 exonuclease activity in the absence of ATP, as seen in Supplementary Fig. S7, the addition of nucleotide caused a statistically significant increase in 2-AP signal for all the mutants when compared to the WT complex (p-value < 0.05). Again, when the relative activity is plotted in the order of NMR peak position, as shown in Fig. 4a *right*, the observed increase in Mre11 exonuclease function correlates strongly with the observed CSPs shown in the 2D ^13^C,^1^H methyl-TROSY spectra in Fig. 2a. Thus, the increase in Rad50^NBD^ activity upon mutation is also seen for Mre11 exonuclease activity.

To quantify the relationship between the shift in population of Rad50^NBD^ structure and dynamics (Figs. 2 and 3) and the changes in M_2_R_2_ activity (Fig. 4a and Supplementary Table S2), we calculated the Pearson’s correlation coefficients (R_P_) of *δ*_*Methyl*_ versus Rad50^NBD^ nucleotide binding, ATP hydrolysis, and dimerization activities as well as M_2_R_2_ exonuclease activity (Supplementary Table S1). 15 of 25 residues in cluster 1 and eight of ten residues in cluster 2 have a meaningful correlation (R_P_ > 0.65) between dynamic changes in structure and ATP hydrolysis, dimerization, and/or exonuclease activities. And 20 of these 23 residues show a meaningful correlation to all three of these activities, underscoring the intimate association between Rad50 ATP-induced dimerization and subsequent ATP hydrolysis and Mre11 exonuclease activity within M_2_R_2_. Interestingly, all of the correlations in cluster 1 are positive, while all the correlations in cluster 2 are negative – a result that emphasizes the power of CHESCA to cluster residues into relevant groups. None of the residues in cluster 3 correlate with these M_2_R_2_ activities (i.e., ATP-induced dimerization, ATP hydrolysis, or exonuclease). The side chain methyl groups of clusters 1 and 2 that are correlated with activity are highlighted on the structure of Mre11^HLH^- Rad50^NBD^ in Fig. 4b. This view shows a path around the perimeter of Rad50^NBD^, from the signature motif to the hinge across the P-loop and down to the Walker A motif, linking the two sites of ATP-binding with the dimerization interface (Supplementary Fig. S8).

## Discussion

Even though X-ray crystallography has revealed the large structural changes that occur to the M_2_R_2_ complex upon Rad50 ATP binding^14–17^, there are still unanswered questions about how this global change and underlying local motions affect DNA DSB recognition, repair initiation, and downstream signaling. High-resolution solution-state studies should be able to provide key information about structures and dynamics not accessible by static crystallography or low-resolution small angle X-ray scattering (SAXS) data. Here, we use methyl-based solution state NMR to further the existing structural biology studies on the essential M_2_R_2_ complex. We coupled our NMR data on Pf Rad50^NBD^ with biochemical data to learn more about a dynamic allosteric network that transmits information throughout Rad50 to promote function.

The basic switch residue R805 was first identified through the comparison of monomer and ATP-induced dimer Mre11^HLH^ -Rad50^NBD^ crystal structures^18^. Subsequent X-ray crystallography, biochemical, and *in vivo* data has shown that the R805E mutant has altered ATP hydrolysis activity and a higher propensity to form dimers in the presence of ATP, which in turn alters M_2_R_2_ DNA end tethering, Mre11 endonuclease activity, and downstream signaling for DNA DSB repair in yeast^19^. The methyl-TROSY ^13^C,^1^H correlation spectrum of Mre11^HLH^ -Rad50^NBD^ (R805E) shows many large CSPs, including regions perturbed in the crystal structure of Pf Rad50^NBD^ (R805E) (i.e., the P-loop) and others that are not (i.e., the base of extended signature helix and β strands 8, 9, and 10). The X-ray crystal structures of WT Rad50^NBD^ (pdb: 3QKS^18^) and Rad50^NBD^(R805E) (pdb: 4NCI^19^) are similar with a Cα and all atom r.m.s.d. of ~1.7 Å and ~2.0 Å, respectively. Yet, our data demonstrates that this basic switch mutation affects every part of Rad50^NBD^ involved in ATP binding and hydrolysis and dimerization. We have also identified two additional mutations, V160M and V156M in the signature-coupling α2 helix, that affect the same residues as R805E in the Mre11 ^HLH^-Rad50^NBD^ methyl correlation spectra. V160 packs against R805, so we hypothesize that the longer side chain of the V160M mutation acts to sterically hinder R805 from its main chain interaction with the carbonyl group of N134. It is therefore not surprising that V160M has similar effects as R805E in the methyl correlation spectra and in the biochemical assays. V156 is on the same face of the signature-coupling α2 helix as V160, but the side chain points toward the signature-coupling α1 helix. We postulate that the V156M mutation begins the process of splaying these two helices apart, which occurs upon ATP binding^18^. Although the movement of these two helices will affect the conserved Q-loop leading to changes in basic switch R805 interactions, the effects would be lesser, resulting in the intermediate changes that we observe in methyl group chemical shift.

The dynamics information supplied by our NMR data greatly enhances the pictures provided by X-ray crystallography. The data presented in Figs 2 and 3 are unique in that many of the CSPs observed with mutation are correlated with a change in the amplitude of side chain methyl group motion. Changes in the salt-bridge and hydrogen bonding network noted by Deshpande *et al*.^19^, and seen in our CSPs (Fig. 2b), have been implicated in the altered activities of the R805E mutation. Here, we show that a general loosening of side chain motions (~65% of residues in the network are experiencing an increase in dynamics – Supplemental Table S1), probed by the build-up of “forbidden” methyl ^1^H triple quantum coherences, also accompanies these previously observed changes in structure. Among the residues with the largest change in *η* rates upon mutation, signifying a change in ${S}_{axis}^{2}$, are L802 and L806 which are in the extended signature helix and line a hydrophobic cavity. The R805E mutation causes a decrease in their *η* rates that corresponds to ${\rm{\Delta }}{S}_{axis}^{2}$, or change in methyl axis order parameters, of ~−0.3–−0.4 (when using an estimated τ_c_ of 30 nsec; see Supporting Information). This is a considerable decrease for a parameter that ranges between 0 and 1 for a completely flexible or rigid methyl group, respectively. Previous biochemical studies have shown that filling this cavity, via a L802W mutation, resulted in decreased ATP binding and hydrolysis and ATP-induced dimerization^19^. The data in Fig. 3 now also suggest a dynamic role for this hydrophobic cavity in addition to a simple structural role.

**Figure 5:**
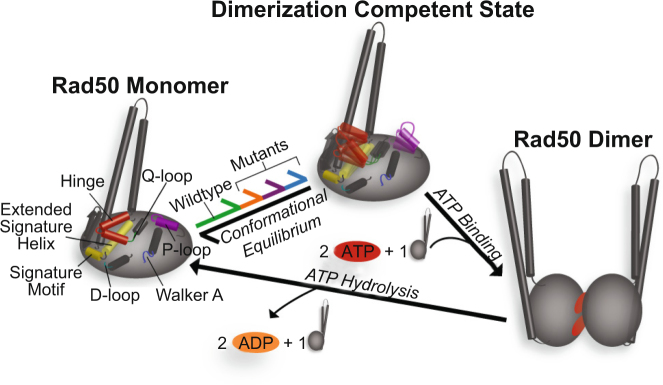
Model for Rad50^NBD^ “activation” to a binding competent state. *Left*, illustrates the “ground” state Rad50^NBD^ with stable interactions between the hinge, extended signature helix, basic switch, and Q-loop. This state is in equilibrium (green reaction arrow) with an ensemble of “Dimerization Competent States,” *middle*, in which the stable interactions are starting to break and increased side chain dynamics are present. The effect of V156M, V160M, and R805E is to increase the population of this state, as denoted by the orange, purple, and blue reaction arrows. ATP binds to this state, and dimerization can subsequently occur, *right*. The process is reset by ATP hydrolysis and dimer dissociation.

While these data provide a description for altered structure and dynamics (i.e., breaking non-covalent interactions and increasing motions) of R805E, V160M, and V156M, these data more importantly provide a different view into the changes in conformation and mobility within Rad50^NBD^ that have to be overcome when ATP binds in order to make a dimer and consequently productive ABC ATPase. Each of these mutations causes many small changes across the structure of Rad50^NBD^ spread across ~50 Å, in contrast to the large global rearrangements that occur upon dimerization, which prepares Rad50^NBD^ for ATP binding and subsequent dimerization. Moreover, these mutations appear to increase conformational entropy, probed through a general increase in methyl group dynamics^40,41^, unlocking dynamics that need to be accessed for ATP binding. These effects are what we observe in the structural and dynamics data presented in Figs 2 and 3. As illustrated in the model in Fig. 5, these structural and dynamics changes appear to lead to a more “active,” dimerization competent Rad50^NBD^ conformation, either through destabilizing the ATP-free ground state and/or increasing the population of this dimerization competent state. The model for the ATP binding competent state in Fig. 5 is simplified as the data in Fig. 2 suggests the presence of more than one excited state. Yet, these states are very likely in a dynamic equilibrium interconverting on a fast timescale, with one (or more) responsible for on-pathway ATP-dependent dimerization. Given the fast exchange on the chemical shift timescale of Fig. 2a and the lack of observable relaxation dispersion CPMG, a lower limit on the observed rate of exchange (*k*_*ex*_) can be placed at ~2000 sec^−1^. Because of the populated weighted average of the fast timescale methyl amplitudes shown in Fig. 3a, it is tempting to place the conformational exchange on the nanosecond timescale; however, these correlations do not arise because exchange is fast relative to *τ*_*c*_ or the timescale of ${S}_{axis}^{2}\,\,$but rather fast relative to the *η* rates, which is actually slower than the CPMG window. Thus, we hypothesize an upper limit on the observed *k*_*ex*_ of ~10^7^ sec^−1^ – a range of conformational exchange rates that have yet to be accessed for methyl groups by NMR spectroscopy. The increased probability of an active Rad50^NBD^ then manifests itself in the observed increases in ATP hydrolysis, Rad50 dimerization, and Mre11 exonuclease activity (Fig. 4).

An allosteric pathway between Mre11 and Rad50 has been implicated in the past with a likely conduit being the Rad50 coiled-coil domain and Mre11^HLH^ interaction^18,19^. However, we did not see any changes in methyl group chemical shift or fast timescale dynamics in this area (Figs 2 and 3) with our mutations. We do observe increased mobility at the base of the coiled-coil domain, which could lead to rigid body motions that would be transmitted to Mre11 via the helix-loop-helix motif^18^, but the NMR data presented here would not be sensitive to such rigid body motions. Furthermore, this route would require transmission of the Rad50 ATP state via a long flexible linker between Mre11^HLH^ and its capping domain. On the other hand, we observe changes in structure and dynamics at the base of the extended signature helix, the signature motif, and β strands 8, 9, and 10. This area of Rad50^NBD^ makes contact with the Mre11 capping domain, a region thought to be important for exonuclease substrate DNA unwinding and translocation^4^. Rad50^NBD^ ATP-induced dimerization and subsequent hydrolysis could power the conformational changes in this domain, which are necessary for efficient M_2_R_2_ nuclease activity. Thus, this second interaction could be an alternative or additional pathway for Mre11-Rad50 allostery. The mutations that we have produced and characterized here are perfect tools for upcoming methyl-based NMR studies on ILVM-labeled Mre11 to pin down the role of the Rad50 ABC ATPase in M_2_R_2_ DNA DSB activity. In conclusion, we have shown that methyl-based NMR methods are sensitive to the subtle structural and dynamics changes that accompany mutation in Rad50^NBD^ and provide a view into the processes that underlie Rad50^NBD^ functions required for DNA DSB repair.

## Materials and Methods

### Protein expression and purification

Codon optimized Pf Rad50 nucleotide binding domain (Rad50^NBD^: aa1-195; GGAGGAGG linker; aa709-882) was synthesized by Life Technologies and subsequently cloned into a modified pET-29 vector (Novagen). Point mutations were made using the Quikchange (Stratagene) approach. *E. coli* BL21(DE3) C41 cells (Sigma) were transformed and grown in deuterated M9 minimal media with 1 g/L ^15^NH_4_Cl and 3 g/L ^2^H,^12^C-glucose. U-[^2^H,^15^N], Ileδ1-[^13^CH_3_], Leuδ/Valγ-[^13^CH_3_,^12^CD_3_], Metε-[^13^CH_3_] (ILVM) labeled samples of 6xHis-Rad50^NBD^ were produced following the protocol of Tugarinov *et al*. with precursors added to the bacterial culture 45 min before induction^42^. Protein expression was induced with 1 mM IPTG for ~16 h at 25 °C. Cells were lysed in 25 mM HEPES, pH 7.0, 300 mM NaCl, 25 mM imidazole via homogenization, and then heated for 30 min at 65 °C. Clarified cell lysate was loaded onto a 5 mL HisTrap HP column (GE Healthcare) and eluted with 300 mM imidazole. The 6xHis tag was removed with TEV protease and Rad50^NBD^ was repurified on the HisTrap column. The sample was subsequently diluted to decrease the NaCl concentration to ~80 mM, then loaded onto a HiTrap SP HP column (GE Healthcare), and eluted in a linear gradient into 25 mM HEPES, pH 7.0, 1 M sodium acetate, 0.1 mM EDTA. The SP elution peak was concentrated using ultrafiltration (Vivaspin, Sartorius) before loading onto a HiLoad 16/60 Superdex 200 pg column (GE Healthcare) equilibrated in 25 mM HEPES, pH 7.0, 200 mM sodium acetate, 0.1 mM EDTA.

Pf Mre11^HLH^ (aa344-426) was sub-cloned from codon optimized full–length Pf Mre11 (synthesized by Life Technologies) into a pETM-60 vector containing an N-terminal NusA-6xHis tag. The plasmid was transformed into *E. coli* BL21(DE3) C41 cells, and protein expression was induced with 1 mM IPTG for 4 h at 37 °C. Cells were lysed in 50 mM HEPES, pH 7.5, 30% (v/v) glycerol via homogenization. Clarified cell lysate was loaded onto a 5 mL HisTrap HP column and eluted with 300 mM imidazole. The NusA-6xHis tag was removed with TEV protease, followed by dialysis into 50 mM HEPES, pH 7.5, 300 mM NaCl, 10% (v/v) glycerol. Mre11^HLH^ was repurified on the HisTrap column. The sample was then brought up to 1.5 M ammonium sulfate and loaded onto a HiTrap Phenyl HP column (GE Healthcare). Mre11^HLH^ was eluted in a step gradient of decreasing ammonium sulfate. A final HisTrap column purification was performed on the 95% B elution peak containing Mre11^HLH^ and Mre11^HLH^ was collected in the flow through.

Mre11^HLH^-Rad50^NBD^ complexes were made by mixing Rad50^NBD^ with a slight excess of Mre11^HLH^ and heating at 60 °C for 10 min. Once cooled, the Mre11^HLH^-Rad50^NBD^ complex was purified on the HiTrap SP HP column. For NMR samples, Mre11^HLH^-Rad50^NBD^ complexes were buffer exchanged into deuterated NMR buffer (25 mM HEPES, pH 7.0 [uncorrected], 150 mM NaCl) and concentrated.

### NMR assignments

Assignments were made using 3D ^13^C,^13^C,^1^H HMQC-NOESY-HMQC data (250 msec mixing time) recorded on a 900 MHz (21.1 T) Varian VNMRS NMR spectrometer equipped with a cryogenically cooled z-axis gradient probe (Rocky Mountain Regional 900 MHz Facility, University of Colorado, Denver). NOE-based assignments were validated and extended by recording 2D ^13^C,^1^H methyl-TROSY HMQC spectra on cysteine point mutations (I190C, V768C, V866C, L103C, L176C, and L51C) in the absence and presence of MTSL nitroxide spin label (Toronto Research Chemicals). Additionally, Rad50 hinge region mutations (V156M, V157M, V160M, and L163M) were produced to help resolve a crowded region in the Rad50^NBD^ structure. The choice of the mutation to methionine was derived from a sequence alignment of Rad50, which showed the need for a hydrophobic residue at these positions, and the desire to put the newly labeled methyl group in an uncrowded region of the methyl-TROSY spectra. HMQC spectra on these mutations were recorded on a 600 MHz (14.1 T) Agilent DD2 NMR spectrometer equipped with a room temperature z-axis gradient probe. All NMR data were collected at 50 °C, and spectra were processed with NMRPipe^43^ and analyzed with CCPN analysis^44^.

### Methyl chemical shift perturbation analysis

To help analyze the effect that mutation has on each of the side chain methyl groups, a combined weighted methyl chemical shift^37^ value (${\delta }_{Methyl}$) was calculated from the ^13^C (*δ*_*C*_) and ^1^H (δ_H_) chemical shifts according to

1 $${\delta }_{Methyl}={\delta }_{C}/{w}_{C}+{\delta }_{H}/{w}_{H}$$

where *w*_*c*_ = (1.65, 1.6, 1.4, and 1.54) and *w*_*H*_ = (0.29, 0.28, 0.27, and 0.41) are the standard deviations for *δ*_*C*_ and *δ*_*H*_ from the Biological Magnetic Resonance Data Bank for Ileδ1, Leuδ, Valγ, and Metε methyl groups, respectively. To determine which methyl groups experience a deviation due to mutation, we used a median-absolute-deviation (MAD) approach^29,30^, which is routinely applied to distributions to find outliers. Here we assume that in the absence of an effect, there will be small, random fluctuations in the peak position due to noise which will be reflected in a small standard deviation. If one or two mutations have an effect then a slightly larger standard deviation will be calculated. Finally, if the mutations have a systematic effect on the peak position, a large standard deviation will be obtained. Thus, for each methyl group, the standard deviation of the four ${\delta }_{Methyl}$ values (i.e., WT, R805E, V160M, and V156M) was determined. We then calculated the median of the ${\delta }_{Methyl}$ standard deviations, and for each methyl group calculated the absolute value of the difference between the median and the methyl group standard deviation. This value along with its median was then converted into a *Z-score* (see Supplementary Fig. S3 for flow chart of analysis). Methyl groups with *Z-scores* greater than 0.25, 1.5, and 2.5 were used to color atoms in Supplementary Fig. S3 light blue, blue, and dark blue respectively.

Chemical shift covariation analysis (CHESCA)^32^ was performed with in-house written python scripts that make use of the scientific python (scipy) library. Using methyl groups that had a significant deviation according to the MAD approach (*Z-score* > 0.25), unit-less inter-residue correlation distances^45,46^ (*d*_*ij*_) were calculated between each side chain methyl group using the ${\delta }_{Methyl}$ values for each mutation according to

2 $${d}_{ij}=1-\frac{({\delta }_{methyl,i}-{\bar{\delta }}_{methyl,i})\cdot ({\delta }_{methyl,j}-{\bar{\delta }}_{methyl,j})}{{\Vert ({\delta }_{methyl,i}-{\bar{\delta }}_{methyl,i})\Vert }_{2}{\Vert ({\delta }_{methyl,j}-{\bar{\delta }}_{methyl,j})\Vert }_{2}}$$

where ${\delta }_{methyl,i}$ (${\delta }_{methyl,j}$) and ${\bar{\delta }}_{methyl,i}$ (${\bar{\delta }}_{methyl,j}$) are a vector and mean of the ${\delta }_{Methyl}$ values for the i-th (j-th) residues, respectively, the · is the dot product, and || ||_2_ is the norm. The correlation distance is similar to the Pearson’s correlation coefficient; however, the correlation distance is zero only if the random vectors (i.e., ${\delta }_{methyl,i}$ and ${\delta }_{methyl,j}$) are truly independent^45,46^. *d*_*ij*_ values were not filtered prior to clustering. Connections were determined using the complete linkage algorithm, which has been shown to minimize false positives compared to the original single linkage method^47^. A unit-less distance cut off of 1.5, which was empirically determined based on the dendrogram in Supplemental Fig. S4, was used to derive the three clusters. The python script to perform CHESCA on methyl residues is available upon request.

### Methyl group relaxation and analysis

The build-up and decay of methyl ^1^H ‘forbidden’ triple-quantum coherences were measured at 50 °C as described by Sun *et al*. using an Agilent 600 MHz (14.1 T) DD2 NMR spectrometer^36^. Relaxation delays (*T*) of 2, 4, 7, 12, 17, 22, 27, 32, 37, and 42 msec were recorded in an interleaved manner. Datasets were processed and analyzed using NMRPipe and associated programs^43^. Intra-methyl ^1^H - ^1^H dipolar cross-correlated relaxation rates ($\eta$) were calculated by fitting ratios of peak intensities as a function of relaxation time,

3 $$|\frac{{I}_{forbid}}{{I}_{allow}}|=\frac{C(\eta \,\tanh (\sqrt{{\eta }^{2}+{\delta }^{2}}T))}{\sqrt{{\eta }^{2}+{\delta }^{2}}-\delta \,\tanh (\sqrt{{\eta }^{2}+{\delta }^{2}}T)}$$

where *I*_*forbid*_ and *I*_*allow*_ are the intensity of the ^1^H triple- and single-quantum coherences, respectively, *C* = 0.75, and *δ* accounts for relaxation from external protons^36^. The reported errors were calculated from the covariance matrix of the fit^48^.

### ATP binding affinity

ATP binding affinity of Mre11^HLH^-Rad50^NBD^ was measured as described previously by Majka *et al*. by observing the fluorescence polarization of BODIPY FL ATP (Adenosine 5′Triphosphate, BODIPY FL 2′-(or-3′)-O-(N-(2-Aminoethyl)-Urethane), Life Technologies) in a Synergy Neo2 multi-mode reader (BioTek)^17^. Reactions were performed in 25 mM HEPES, pH 7.0, 150 mM NaCl, 0.1 mM EDTA. Mre11^HLH^-Rad50^NBD^ (0–70 μM) was titrated into a black, flat bottom 384-well plate where each well contained 5 nM BODIPY FL ATP and 5 mM MgCl_2_. The plate was centrifuged at 500 × g for 2 min and then incubated at room temperature for 10 min. Parallel and perpendicular fluorescence were measured using a FP 485/530 filter; polarizations were calculated via BioTek software. Binding affinities were calculated by fitting polarization (*F*) vs. protein concentration ([*P*]) with an in-house script to the quadratic binding function,

4 $$F={F}_{0}+({F}_{Max}-{F}_{0})\frac{({K}_{D}+[ATP]+[P])-\sqrt{{({K}_{D}+[ATP]+[P])}^{2}-4[ATP][P]}}{2\ast [ATP]}$$

where *F*_0_ and *F*_*Max*_ are initial and final polarization values. Reported errors are the standard deviation of n ≥ 3 experiments.

### ADP binding affinity

Mre11^HLH^-Rad50^NBD^ binding affinity to ADP was measured using FRET between the intrinsic tryptophan residues in Rad50 and MANT-ADP (2′-(or-3′)-*O*-(*N*-Methylanthraniloyl) Adenosine 5′-Diphosphate, Thermo Scientific). Reactions were performed in 25 mM HEPES, pH 7.0, 150 mM NaCl, 0.1 mM EDTA. MANT-ADP was titrated (0–50 µM) into a black, flat-bottom 384-well plate, where each well contained 5 µM Mre11^HLH^-Rad50^NBD^ and 5 mM MgCl_2_. The plate was then centrifuged at 500 × g for 2 min and incubated at room temperature for 10 min. Tryptophan residues were excited at 290 nm and MANT emission from FRET was detected at 450 nm on the Synergy Neo2 plate reader. Spectral overlap was corrected using the tryptophan emission from an unlabeled ADP titration with the appropriate protein concentration and the emission from a titration of MANT-ADP alone, where the signal from FRET has the background contribution from donor and acceptor subtracted from each point^49^. Binding affinities were calculated by fitting FRET intensity vs. protein concentration to the quadratic binding function equation (4). Reported errors are the standard deviation of n ≥ 3 experiments.

### Nucleotide competition studies

Binding affinities for Mre11^HLH^-Rad50^NBD^ to ATP and ADP were also measured via a competition assay, as shown for the data in Supplementary Fig. S6. BODIPY FL ATP was pre-bound to Mre11^HLH^-Rad50^NBD^ (wildtype or R805E) to achieve ~80% fraction bound, and a decrease in fluorescence polarization was monitored as a function of increasing unlabeled ATP or ADP (Supplementary Fig. S6). Reactions were performed in 25 mM HEPES, pH 7.0, 150 mM NaCl, and 0.1 mM EDTA. ATP or ADP (0–1 mM) was titrated into a black, flat bottom 384-well plate where each well contained 5 nM BODIPY FL ATP, 5 mM MgCl_2_, and 25 μM Mre11^HLH^-Rad50^NBD^ (wildtype or R805E). The plate was then centrifuged at 500 × g for 2 min and then incubated at room temperature for 10 min. Parallel and perpendicular fluorescence were measured using a Synergy Neo2 multimode reader equipped with a FL 485/530 filter (Biotek); polarizations were calculated via BioTek software. Binding affinities were calculated by fitting polarization vs. unlabeled nucleotide concentration with an in-house script to the previously described inhibition function competitive binding function^50^. The calculated K_I,ATP_ and K_I,ADP_ are in agreement with the data presented in Supplementary Table S2, showing the same trends as observed for the K_D_’s determined with the fluorescently labeled nucleotide analogs.

### Dimerization

Mre11^HLH^-Rad50^NBD^ dimerization was analyzed on a Superdex 200 Increase 10/300 GL column (GE Healthcare) as described by Desphande *et al*.^19^. Each sample contained 75 μM Mre11^HLH^-Rad50^NBD^ and 5 mM MgCl_2_ in 50 μL with and without 5 mM ATP. Samples were incubated at 60 °C for 10 min, cooled briefly on ice, and centrifuged for 10 min at 14,000 rpm. The samples were then loaded onto the column equilibrated in 25 mM HEPES, pH 7.0, 150 mM NaCl, and 0.1 mM EDTA at 4 °C. The fraction of dimeric Mre11^HLH^-Rad50^NBD^ was determined by measuring the area under the curve, using a box sum, for monomer and dimer peaks and dividing the area of the dimer by the total area of the two peaks.

### ATP hydrolysis assay

ATP hydrolysis kinetics were measured in 50 mM Tris, pH 7.75, 100 mM NaCl, and 10 mM MgCl_2_. 60 μL reactions containing either 0.5 μM (for V160M and R805E) or 2 μM Mre11_2_-Rad50^NBD^_2_ (For wildtype and V156M) with 1.2-fold excess Mre11 and 0–150 μM ATP were incubated for 60 min at 65 °C, then cooled on the bench for 2 min before centrifuging at max speed for 1 min. Reactions without protein at each ATP concentration (i.e., an ATP only titration) were included for each experiment as a control for ATP degradation/PO_4_ contamination. 50 µL of each reaction was transferred into clear, flat-bottom 96-well plates, then 100 µL of cold BIOMOL Green reagent (Enzo Life Sciences) was added to each well. The plates were centrifuged at 500 × g for 1 min and the BIOMOL reaction was allowed to proceed at room temperature for 30 min. The plate was read for BIOMOL Green signal at A_640_ in the Synergy Neo2 multi-mode reader using the path length correction function to correct for any differences in volumes between the wells. A_640_ values were corrected by subtracting the A_640_ values of the ATP only reactions at each ATP concentration. This corrected A_640_ value was then transformed into nmols of PO_4_ released/min based on a PO_4_ standard curve incubated in BIOMOL Green reagent for 30 min at room temperature. These values (*v*_0_) were plotted versus ATP concentration and the data were fit to the Michaelis-Menten equation including a Hill coefficient (*n*)

5 $${v}_{0}=\frac{{V}_{max}\,{[ATP]}^{n}}{{K}_{M}^{n}+{[ATP]}^{n}}$$

to find the *V*_*max*_ and *K*_*M*_. Plots are the average of least 3 replicate experiments.

### Mre11 exonuclease assay

The 2-aminopurine (2-AP) exonuclease assays were performed in 50 mM Tris pH 7.5, 150 mM NaCl, 0.1% PEG-6000, and 2.5% glycerol as described by Williams *et al*.^4^. Two DNA strands Exo2-5 (5′-GGCGTGCCTTGGGCGCGCTGCGGGCGG(2-AP)G-3′) and Exo2-3 (5′-CTCCGCCCGCAGCGCGCCCAAGGCACGCC-3′) were resuspended in 10 mM Tris, pH 7.5 at a concentration of 100 μM. Equal volumes of strands were combined, heated for 5 min at 95 °C, then slowly cooled to room temperature to allow the strands to anneal. 30 μL exonuclease reactions contained 0.5 μM Mre11_2_-Rad50^NBD^_2_ complex (with 1.2-fold excess Rad50^NBD^) and 1 μM 2-AP DNA duplex plus 1 mM MnCl_2_ or 1 mM MnCl_2_/5 mM MgCl_2_/1 mM ATP or 1 mM MnCl_2_/5 mM MgCl_2_/1 mM AMP-PNP. Reactions were incubated at 60 °C for 45 min in a heat block (and in the dark to prevent quenching of the fluorophore), then cooled on the bench for 2 min before centrifuging at max speed for 1 min. 25 μL of each reaction was transferred to black, flat-bottom 384-well plates. The plates were centrifuged at 500 × g for 1 min and then 2-AP fluorescence (ex310/em375) was read in a Synergy Neo2 plate reader. Raw relative fluorescent units were corrected by subtracting the signal from a reaction without MnCl_2_ or nucleotide. At least 3 replicate experiments were done for each mutant Mre11_2_-Rad50^NBD^_2_ complex across at least two preparations of complex.

### Data availability

The datasets generated during and/or analysed during the current study are available from the corresponding author on reasonable request.

## Electronic supplementary material

Supplemental Information

## References

[1] Rupnik A, Lowndes NF, Grenon M (2010). MRN and the race to the break. Chromosoma.

[2] Williams RS, Williams JS, Tainer JA (2007). Mre11-Rad50-Nbs1 is a keystone complex connecting DNA repair machinery, double-strand break signaling, and the chromatin template. Biochem. Cell Biol..

[3] Paull TT, Gellert M (1998). The 3′ to 5′ Exonuclease Activity of Mre11 Facilitates Repair of DNA Double-Strand Breaks. Mol. Cell.

[4] Williams RS (2008). Mre11 dimers coordinate DNA end bridging and nuclease processing in double-strand-break repair. Cell.

[5] Hopfner K-P (2000). Structural biology of Rad50 ATPase: ATP-driven conformational control in DNA double-strand break repair and the ABC-ATPase superfamily. Cell.

[6] Hopfner K-P, Tainer JA (2003). Rad50/SMC proteins and ABC transporters: Unifying concepts from high-resolution structures. Curr. Opin. Struct. Biol..

[7] Lee J-H, Paull TT (2007). Activation and regulation of ATM kinase activity in response to DNA double-strand breaks. Oncogene.

[8] Williams RS (2009). Nbs1 flexibly tethers Ctp1 and Mre11-Rad50 to coordinate DNA double-strand break processing and repair. Cell.

[9] Carney JP (1998). The hMre11/hRad50 protein complex and Nijmegen breakage syndrome: Linkage of double-strand break repair to the cellular DNA damage response. Cell.

[10] Waltes R (2009). Human RAD50 Deficiency in a Nijmegen Breakage Syndrome-like Disorder. Am. J. Hum. Genet..

[11] Stewart GS (1999). The DNA double-strand break repair gene hMRE11 is mutated in individuals with an ataxia-telangiectasia-like disorder. Cell.

[12] Wang Z (2004). Three Classes of Genes Mutated in Colorectal Cancers with Chromosomal Instability. Cancer Res..

[13] Vilar E (2011). MRE11 deficiency increases sensitivity to poly(ADP-ribose) polymerase inhibition in microsatellite unstable colorectal cancers. Cancer Res..

[14] Möckel C, Lammens K, Schele A, Hopfner K-P, Pemberton T (2012). ATP driven structural changes of the bacterial Mre11:Rad50 catalytic head complex. Nucleic Acids Res..

[15] Lammens K (2011). TheMre11:Rad50 structure shows an ATP-dependent molecular clamp in DNA double-strand break repair. Cell.

[16] Lim HS, Kim JS, Park YB, Gwon GH, Cho Y (2011). Crystal structure of the Mre11-Rad50-ATPγS complex: understanding the interplay between Mre11 and Rad50. Genes Dev..

[17] Majka J, Alford B, Ausio J, Finn RM, McMurray CT (2012). ATP hydrolysis by RAD50 protein switches MRE11 enzyme from endonuclease to exonuclease. J. Biol. Chem..

[18] Williams GJ (2011). ABC ATPase signature helices in Rad50 link nucleotide state to Mre11 interface for DNA repair. Nat. Struct. Mol. Biol..

[19] Deshpande RA (2014). ATP-driven Rad50 conformations regulate DNA tethering, end resection, and ATM checkpoint signaling. EMBO J..

[20] Hopkins BB, Paull TT (2008). The P. furiosus mre11/rad50 complex promotes 5′ strand resection at a DNA double-strand break. Cell.

[21] Seifert FU, Lammens K, Stoehr G, Kessler B, Hopfner K-P (2016). Structural mechanism of ATP-dependent DNA binding and DNA end bridging by eukaryotic Rad50. EMBO J..

[22] Hohl M (2015). Interdependence of the Rad50 Hook and Globular domain functions. Mol. Cell.

[23] Gao Y, Meyer JR, Nelson SW (2016). A network of allosterically coupled residues in the bacteriophage T4 Mre11-Rad50 complex. Protein Sci..

[24] Pervushin K, Riek R, Wider G, Wüthrich K (1997). Attenuated T2 relaxation by mutual cancellation of dipole-dipole coupling and chemical shift anisotropy indicates an avenue to NMR structures of very large biological macromolecules in solution. Proc. Natl. Acad. Sci. USA.

[25] Tugarinov V, Hwang PM, Ollerenshaw JE, Kay LE (2003). Cross-Correlated Relaxation Enhanced 1 H− 13 C NMR Spectroscopy of Methyl Groups in Very High Molecular Weight Proteins and Protein Complexes. J. Am. Chem. Soc..

[26] Sprangers R, Kay LE (2007). Quantitative dynamics and binding studies of the 20S proteasome by NMR. Nature.

[27] Venditti, V., Fawzi, N. L. & Clore, G. M. Automated sequence- and stereo-specific assignment of methyl-labeled proteins by paramagnetic relaxation and methyl-methyl nuclear overhauser enhancement spectroscopy. *J. Biomol. NMR*, 10.1007/s10858-011-9559-4 (2011). 10.1007/s10858-011-9559-4 PMC3212433 21935714

[28] Battiste JL, Wagner G (2000). Utilization of site-directed spin labeling and high-resolution heteronuclear nuclear magnetic resonance for global fold determination of large proteins with limited nuclear overhauser effect data. Biochemistry.

[29] Pham-Gia T, Hung TL (2001). The mean and median absolute deviations. Math. Comput. Model..

[30] Leys C, Ley C, Klein O, Bernard P, Licata L (2013). Detecting outliers: Do not use standard deviation around the mean, use absolute deviation around the median. J. Exp. Soc. Psychol..

[31] Boulton S, Melacini G (2016). Advances in NMR Methods to Map Allosteric Sites: From Models to Translation. Chem. Rev..

[32] Selvaratnam R, Chowdhury S, Vanschouwen B, Melacini G (2011). Mapping allostery through the covariance analysis of NMR chemical shifts. Proc. Natl. Acad. Sci. USA.

[33] Selvaratnam R, Mazhab-Jafari MT, Das R, Melacini G (2012). The Auto-Inhibitory Role of the EPAC Hinge Helix as Mapped by NMR. PLoS One.

[34] Akimoto M (2013). Signaling through dynamic linkers as revealed by PKA. Proc. Natl. Acad. Sci. USA.

[35] Palmer AG, Kroenke CD, Loria JP (2001). Nuclear magnetic resonance methods for quantifying microsecond-to-millisecond motions in biological macromolecules. Methods Enzymol..

[36] Sun H, Kay LE, Tugarinov V (2011). An optimized relaxation-based coherence transfer NMR experiment for the measurement of side-chain order in methyl-protonated, highly deuterated proteins. J. Phys. Chem. B.

[37] Ådén J, Wolf-Watz M, Adén J, Wolf-Watz M (2007). NMR identification of transient complexes critical to adenylate kinase catalysis. J. Am. Chem. Soc..

[38] Hopfner K-P (2001). Structural biochemistry and interaction architecture of the DNA double-strand break repair Mre11 nuclease and Rad50-ATPase. Cell.

[39] Herdendorf TJ, Albrecht DW, Benkovic SJ, Nelson SW (2011). Biochemical characterization of bacteriophage T4 Mre11-Rad50 complex. J. Biol. Chem..

[40] Frederick KK, Marlow MS, Valentine KG, Wand AJ (2007). Conformational entropy in molecular recognition by proteins. Nature.

[41] Tzeng S-R, Kalodimos CG (2012). Protein activity regulation by conformational entropy. Nature.

[42] Tugarinov V, Kanelis V, Kay LE (2006). Isotope labeling strategies for the study of high-molecular-weight proteins by solution NMR spectroscopy. Nat. Protoc..

[43] Delaglio F (1995). A multidimensional spectral processing system based on pipes. J. Biomol. NMR.

[44] Vranken WF (2005). The CCPN data model for NMR spectroscopy: development of a software pipeline. Proteins.

[45] Székely GJ, Rizzo ML, Bakirov NK (2007). Measuring and testing dependence by correlation of distances. Ann. Stat..

[46] Székely GJ, Rizzo ML (2009). Brownian distance covariance. Ann. Appl. Stat..

[47] Boulton S, Akimoto M, Selvaratnam R, Bashiri A, Melacini G (2014). A tool set to map allosteric networks through the NMR chemical shift covariance analysis. Sci. Rep..

[48] Press, W. H., Flannery, B. P., Teukolsky, S. A. & Vetterling, W. T. *Numerical Recipes in C: The Art of Scientific Computing*. (Cambridge University Press, 1992).

[49] Hieb AR (2012). Fluorescence strategies for high-throughput quantification of protein interactions. Nucleic Acids Res..

[50] Wang ZX (1995). An exact mathematical expression for describing competitive binding of two different ligands to a protein molecule. FEBS Lett..

