# Supplementary material for: A dynamic allosteric pathway underlies Rad50 ABC ATPase function in DNA repair
Source: Sci Rep. 2018 Jan 26;8:1639. doi: 10.1038/s41598-018-19908-8 (PMC5786021; doi:10.1038/s41598-018-19908-8)
Supplement: Supplementary file 1 — Supplemental Information [file 41598_2018_19908_MOESM1_ESM.pdf]

A dynamic allosteric pathway underlies Rad50 ABC ATPase function in DNA repair

Zachary K. Boswell[a], Samiur Rahman[a], Marella D. Canny[a], and Michael P. Latham[a,1]

[a]Department of Chemistry and Biochemistry, Texas Tech University, Lubbock, TX, USA 79423

[1]Corresponding Author:
Michael P. Latham
Department of Chemistry and Biochemistry
Texas Tech University
Chemistry and Biochemistry Building
1204 Boston Ave.
Lubbock, TX 79409-1061
USA
E-mail: michael.latham@ttu.edu

SUPPORTING INFORMATION

The $Mre11^{HLH}$ domain eliminates a non-native dimer in $Rad50^{NBD}$

Of note, our initial NMR studies began with isolated *P. furiosus* $Rad50^{NBD}$, which produced relatively high quality methyl-TROSY correlation spectra. While the majority of the side chain methyl group assignments could be obtained from these data, several peaks were not observed, and through the process of elimination, the missing data was mapped to the coiled-coil region where $Mre11^{HLH}$ binds. Analysis of side chain methyl group η relaxation rates[1] (Supplementary Fig. S9) provided an estimated rotational correlation time of ~45 nsec, which was in poor agreement with a calculated correlation time of ~26 nsec obtained from HYDROPRONMR[2] and a monomeric $Rad50^{NBD}$ crystal structure (pdb: 1II8[3]). As illustrated in Supplementary Fig. S9 (filled symbols), this sample also showed the presence of msec timescale dynamics as measured by ^{13}C single-quantum Carr-Purcell-Meiboom-Gill relaxation dispersion (^{13}C CPMG RD) experiments[4,5]. However, these dynamics could not be modulated by the mutations described herein or through substrate or product (i.e., non- hydrolysable ATP analog or ADP) binding. As discussed in the main text and shown in Supplementary Fig. S5, we used size exclusion chromatography to monitor the ATP-dependent dimerization of $Rad50^{NBD}$. Under a variety of conditions, such as varying protein and/or ATP concentrations, buffer composition, and temperature, the ATP-dependent shift to dimeric $Rad50^{NBD}$ could not be observed. Also, under every condition used for size exclusion chromatography or NMR, isolated $Rad50^{NBD}$ precipitated in the presence of ATP. Together these results implied that isolated Pf $Rad50^{NBD}$ is in a non-native state recalcitrant to ATP-induced dimerization.

As shown by Tainer and co-workers, the M_2R_2 complex is formed through hydrophobic interactions between the base of the Rad50 coiled-coil domain, present in our construct, and the C-terminal $Mre11^{HLH}$ domain[6]. We hypothesized that similar hydrophobic interactions which mediate M_2R_2 complex formation could be leading to the formation of a non-native $Rad50^{NBD}$ dimer in the absence of Mre11. Indeed, such an interaction was observed in the crystal structures of isolated *M. jannaschii* $Rad50^{NBD}$[7]. The complex of unlabeled $Mre11^{HLH}$ and ILVM-labeled $Rad50^{NBD}$ showed additional peaks in the methyl-TROSY HMQC spectra, which based on conservative mutations were unequivocally assigned to the coiled-coil domain of $Rad50^{NBD}$

where $Mre11^{HLH}$ binds. Moreover, analysis of methyl group triple quantum η relaxation rates revealed a rotational correlation time of ~30 nsec, in very good agreement with calculated value for monomeric $Rad50^{NBD}$, while Supplementary Fig. S9 (open symbols) shows a complete quenching of msec timescale dynamics. Thus, the NMR results suggest the presence of a non-native dimer of $Rad50^{NBD}$ in the absence of $Mre11^{HLH}$, which undergoes conformational exchange on the msec timescale to a monomer state or a conformation forming higher order oligomers with other non-native dimers in solution.

Finally, it is worth noting that the CSPs observed in Fig. 2a were also observed in isolated $Rad50^{NBD}$ in the absence of $Mre11^{HLH}$, indicating that this is a general property of $Rad50^{NBD}$ and not dependent on $Mre11^{HLH}$ binding.

SUPPORTING INFORMATION REFERENCES

1. Sun, H., Kay, L. E. & Tugarinov, V. An optimized relaxation-based coherence transfer NMR experiment for the measurement of side-chain order in methyl-protonated, highly deuterated proteins. *J. Phys. Chem. B* **115,** 14878–84 (2011).
2. García De La Torre, J., Huertas, M. L. & Carrasco, B. Calculation of hydrodynamic properties of globular proteins from their atomic-level structure. *Biophys. J.* **78,** 719–30 (2000).
3. Hopfner, K.-P. *et al.* Structural biology of Rad50 ATPase: ATP-driven conformational control in DNA double-strand break repair and the ABC-ATPase superfamily. *Cell* **101,** 789–800 (2000).
4. Palmer, A. G., Kroenke, C. D. & Loria, J. P. Nuclear magnetic resonance methods for quantifying microsecond-to-millisecond motions in biological macromolecules. *Methods Enzymol.* **339,** 204–38 (2001).
5. Lundström, P., Vallurupalli, P., Religa, T. L., Dahlquist, F. W. & Kay, L. E. A single-quantum methyl 13C-relaxation dispersion experiment with improved sensitivity. *J. Biomol. NMR* **38,** 79–88 (2007).
6. Williams, G. J. *et al.* ABC ATPase signature helices in Rad50 link nucleotide state to Mre11 interface for DNA repair. *Nat. Struct. Mol. Biol.* **18,** 423–31 (2011).
7. Lim, H. S., Kim, J. S., Park, Y. B., Gwon, G. H. & Cho, Y. Crystal structure of the Mre11-Rad50-ATPγS complex: understanding the interplay between Mre11 and Rad50. *Genes Dev.* **25,** 1091–104 (2011).
8. Tugarinov, V., Hwang, P. M., Ollerenshaw, J. E. & Kay, L. E. Cross-Correlated Relaxation Enhanced 1 H− 13 C NMR Spectroscopy of Methyl Groups in Very High Molecular Weight Proteins and Protein Complexes. *J. Am. Chem. Soc.* **125,** 10420–10428 (2003).
9. Battiste, J. L. & Wagner, G. Utilization of site-directed spin labeling and high-resolution heteronuclear nuclear magnetic resonance for global fold determination of large proteins with limited nuclear overhauser effect data. *Biochemistry* **39,** 5355–65 (2000).
10. Venditti, V., Fawzi, N. L. & Clore, G. M. Automated sequence- and stereo-specific assignment of methyl-labeled proteins by paramagnetic relaxation and methyl-methyl

nuclear overhauser enhancement spectroscopy. *J. Biomol. NMR* (2011). doi:10.1007/s10858-011-9559-4

11. Koroleva, O., Makharashvili, N., Courcelle, C. T., Courcelle, J. & Korolev, S. Structural conservation of RecF and Rad50: implications for DNA recognition and RecF function. *EMBO J.* **26,** 867–77 (2007).

12. Wang, Z. X. An exact mathematical expression for describing competitive binding of two different ligands to a protein molecule. *FEBS Lett.* **360,** 111–114 (1995).

Supplementary Table S1. Chemical Shift Perturbation Correlations to Activity

Cluster 1[c]	η^a	$\lvert\Delta\eta\rvert > 8^b$	*ATP Binding*[a]	*ADP Binding*[a]	*ATP Hydrolysis*[a]	*Dimerization*[a]	*Exonuclease*[a]
**800LeuCδ2**	-0.13	No	-0.481	-0.025	0.849	0.797	0.834
**47LeuCδ1**	-0.103	No	-0.657	0.05	0.904	0.953	0.988
**810LeuCδ2**	0.105	Yes	-0.561	0.137	0.945	0.907	0.972
**745LeuCδ2**	-0.744	No	-0.599	-0.107	0.829	0.869	0.877
**851IleCdδ1**	-0.518	No	-0.402	-0.155	0.707	0.663	0.672
**792LeuCδ1**	0.151	No	-0.736	-0.273	0.736	0.913	0.869
**778LeuCδ2**	-0.934	No	-0.526	0.272	0.958	0.891	0.983
**778LeuCδ1**	-0.627	No	-0.741	-0.001	0.852	0.983	0.995
**131IleCδ1**	-0.965	Yes	-0.603	-0.095	0.839	0.877	0.888
802LeuCδ2	-0.721	No	-0.216	-0.502	0.171	0.238	0.152
**161LeuCδ2**	-0.941	No	-0.274	0.334	0.974	0.722	0.854
**45ValCγ1**	-0.54	No	-0.827	-0.104	*0.764*	0.995	0.971
**56IleCδ1**	0.29	Yes	-0.657	-0.416	0.588	*0.777*	0.701
**792LeuCδ2**	-0.132	No	-0.879	-0.458	0.583	0.93	0.823
6ValCγ2	-0.877	No	-0.106	0.095	0.727	0.467	0.551
820IleCδ1	-0.912	No	-0.048	-0.06	0.536	0.321	0.365
806LeuCδ2	-0.093	Yes	-0.495	-0.376	0.546	0.639	0.584
147LeuCδ2	0.323	No	-0.511	-0.789	0.011	0.358	0.174
**19ValCγ2**	-0.067	No	-0.487	-0.266	0.651	0.692	0.667
73LeuCδ1	0.679	No	-0.528	-0.456	0.478	0.625	0.545
163LeuCδ1	-0.903	Yes	-0.973	-0.677	0.305	0.845	0.66
812LeuCδ1	-0.018	Yes	-0.295	-0.148	0.644	0.554	0.568
847IleCδ1	0.326	No	-0.475	-0.643	0.205	0.439	0.304
133LeuCδ1	-0.363	No	0.097	-0.215	0.219	0.047	0.047
**789LeuCδ1**	0.79	No	-0.919	-0.294	0.635	0.989	0.909

Cluster 2[c]	η^a	$\lvert\Delta\eta\rvert > 8^b$	*ATP Binding*[a]	*ADP Binding*[a]	*ATP Hydrolysis*[a]	*Dimerization*[a]	*Exonuclease*[a]
**819LeuCδ2**	-0.295	No	0.448	0.098	*-0.78*	-0.736	*-0.757*
**769ValCγ2**	0.486	Yes	0.607	0.217	*-0.75*	-0.83	-0.811
**146IleCδ1**	0.979	No	0.464	0.095	*-0.79*	*-0.752*	*-0.773*
**136IleCδ1**	-0.861	No	0.444	0.203	-0.687	-0.681	-0.675
**808MetCε**	-0.871	Yes	0.552	-0.036	-0.906	-0.878	-0.923
789LeuCδ2	-0.569	Yes	0.886	*0.777*	0.181	-0.52	-0.275
**748IleCδ1**	*0.756*	No	0.59	-0.135	-0.939	-0.924	-0.985
133LeuCδ2	-0.22	No	0.906	*0.765*	0.137	-0.558	-0.318
**157ValCγ1**	0.67	No	0.682	0.104	-0.842	-0.935	-0.935
**776ValCγ2**	0.028	No	0.849	0.158	-0.745	-0.999	-0.962

Cluster 3[c]	η^a	$\lvert\Delta\eta\rvert > 8^b$	*ATP Binding*[a]	*ADP Binding*[a]	*ATP Hydrolysis*[a]	*Dimerization*[a]	*Exonuclease*[a]
800LeuCδ1	0.214	No	-0.545	-0.262	-0.239	0.255	0.125
859LeuCδ1	-0.648	Yes	0.829	0.598	-0.445	-0.819	-0.679
819LeuCδ1	-0.274	No	-0.017	0.689	0.438	0.257	0.415
821LeuCδ1	-0.98	No	0.252	0.615	-0.043	-0.193	-0.07
767ValCγ1	0.092	Yes	0.477	0.847	0.123	-0.261	-0.057
769ValCγ1	-0.957	No	0.047	0.816	0.82	0.418	0.647
802LeuCδ1	0.243	Yes	0.698	0.722	-0.242	-0.615	-0.447
3LeuCδ1	0.847	No	0.098	0.028	-0.61	-0.397	-0.45
138IleCδ1	0.494	No	0.489	0.503	-0.397	-0.553	-0.461
44LeuCδ2	-0.257	Yes	0.311	0.429	-0.352	-0.404	-0.339
51LeuCδ1	*0.745*	No	-0.131	0.057	-0.37	-0.105	-0.152
806LeuCδ1	0.68	No	0.336	0.473	-0.317	-0.402	-0.323
147LeuCδ1	0.995	No	0.389	*0.75*	0.034	-0.248	-0.079
44LeuCδ1	-0.64	No	-0.122	0.484	0.199	0.2	0.287
812LeuCδ2	0.274	No	-0.659	-0.248	-0.065	0.429	0.308
40LeuCδ1	0.981	No	0.715	0.645	-0.354	-0.688	-0.543
821LeuCδ1	-0.98	No	0.252	0.615	-0.043	-0.193	-0.07

[a]Pearson's correlation coefficient between the methyl group CSP (δ_{methyl}) and the indicated activity. [b]Denotes if the methyl group has a small CSP (range $\Delta\delta_{methyl}$ < 0.13 ppm) but undergoes a large change in dynamics ($|\eta_{WT}$ – the average $\eta_{mutants}| > 8$ sec^{-1}) upon mutation. [c]Methyl

groups in bold font signify residues for which ATP hydrolysis, dimerization, and Mre11 exonuclease activities are strongly correlated to CSP (|mean[$R_{P,Hydrolysis}$, $R_{P,Dimerization}$, $R_{P,Exonuclease}$]| > 0.65). Atoms in bold are also shown on the structures in Fig. 4b and Fig. S8.

Supplementary Table S2. Summary of Rad50 Activity

	$K_{D,\,ATP}$ (μM)[a]	$K_{D,\,ADP}$ (μM)[a]	K_M (μM)[a]	k_{cat} (s^{-1})[a]	Dimerization (% Dimer)[b]
Wildtype	16.9 ± 3.9	2.3 ± 0.2	23.6 ± 1.5	4.7 ± 0.2	38.6
V156M	7.6 ± 0.8***	0.7 ± 0.3***	16.5 ± 0.8**	4.5 ± 0.1	64.1
V160M	12.7 ± 2.8*	4.1 ± 0.2***	13.0 ± 0.5***	22.3 ± 0.5***	66.6
R805E	7.7 ± 1.2***	1.2 ± 0.3**	11.8 ± 0.5***	19.5 ± 0.3***	78.9

[a]Values are the mean ± the standard deviation of at least three measurements from at least two preparations of wildtype or mutant Rad50^{NBD}. *, **, and *** represent p-values less than 0.05, 0.01, and 0.001, respectively. [b] % Dimer is reported from one experiment.

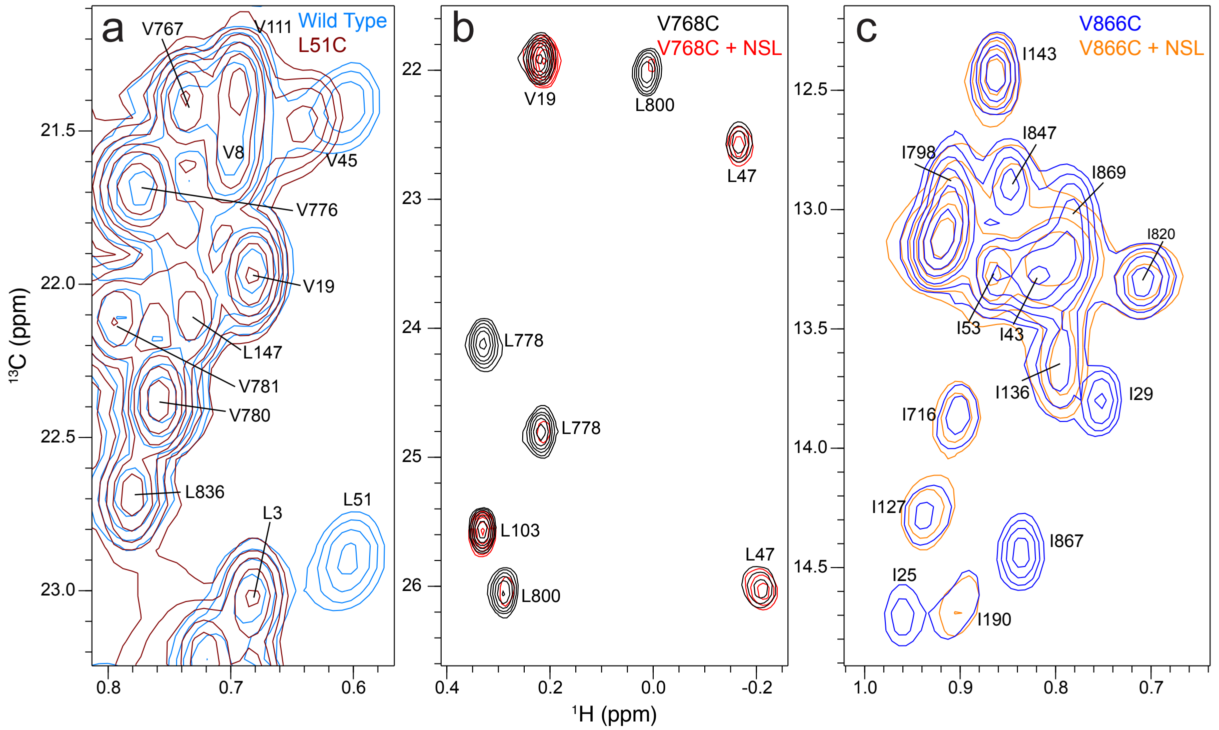

Supplementary Figure S1 | Mutations and paramagnetic relaxation enhancement assisted in assignments. 2D ^{13}C,^{1}H methyl-TROSY HMQC[8] spectra of Mre11^{HLH}-ILVM-labeled Rad50^{NBD} mutants recorded at 14.1 T and 50 °C. Methyl assignments are indicated. (**a**) The L51C mutation removes leucine resonances (i.e., Cδ1 and Cδ2) from the spectrum, while also causing CSPs in nearby residues (e.g.V45). (**b**) V768C and (**c**) V866C spectra shown before and after modification with a MTSL paramagnetic spin label on the introduced cysteine residue[9,10]. Peaks that disappear or have lower intensity experience a much faster relaxation rate due to the proximity of the unpaired electron.

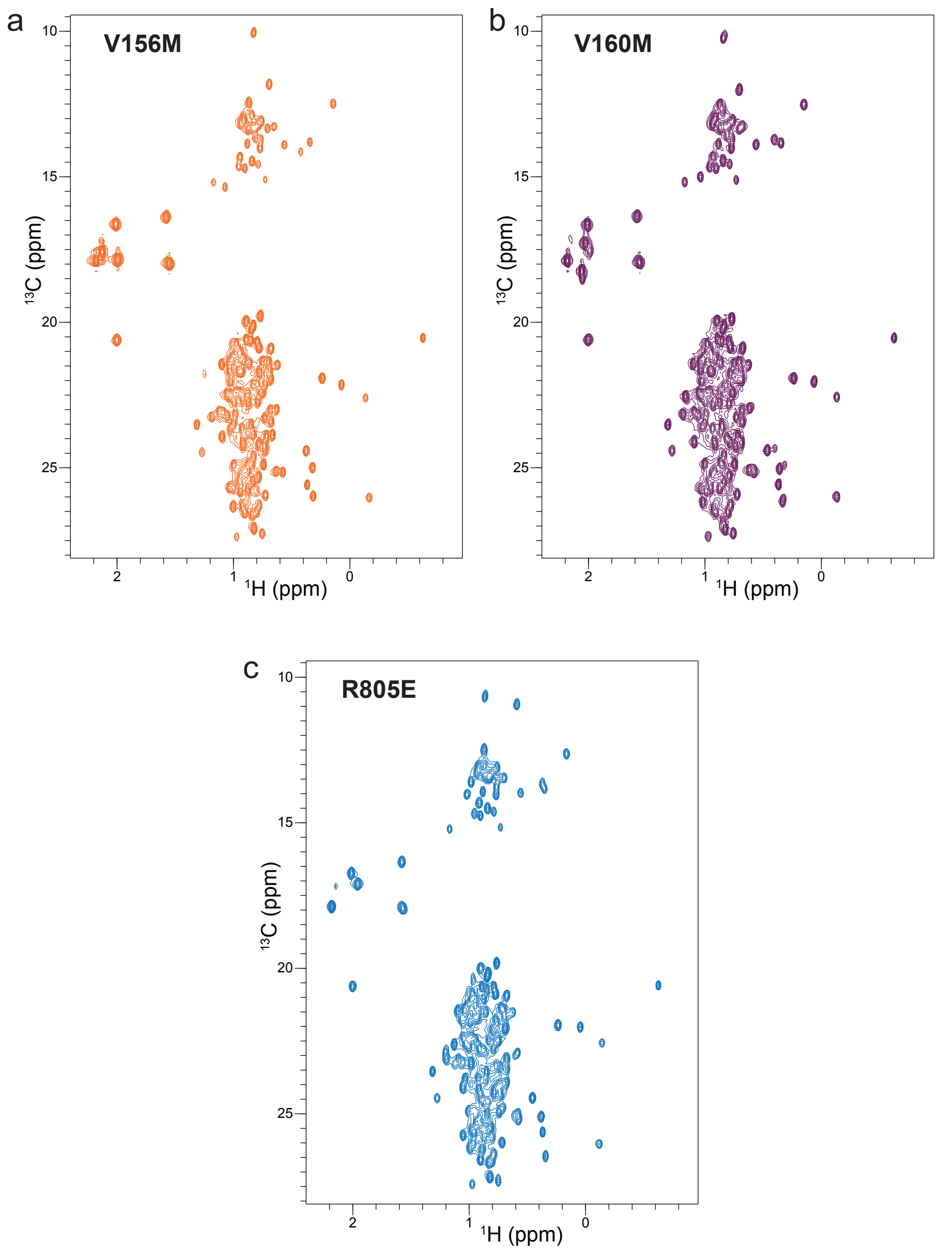

Supplementary Figure S2 | Spectra of basic switch and hinge region Mre11^{HLH}-Rad50^{NBD} mutants. 2D ^{13}C,^{1}H methyl-TROSY HMQC spectra of Mre11^{HLH}-ILVM-labeled Rad50^{NBD} mutants collected at 14.1 T and 50 °C for (**a**) R805E, (**b**) V160M, and (**c**) V156M.

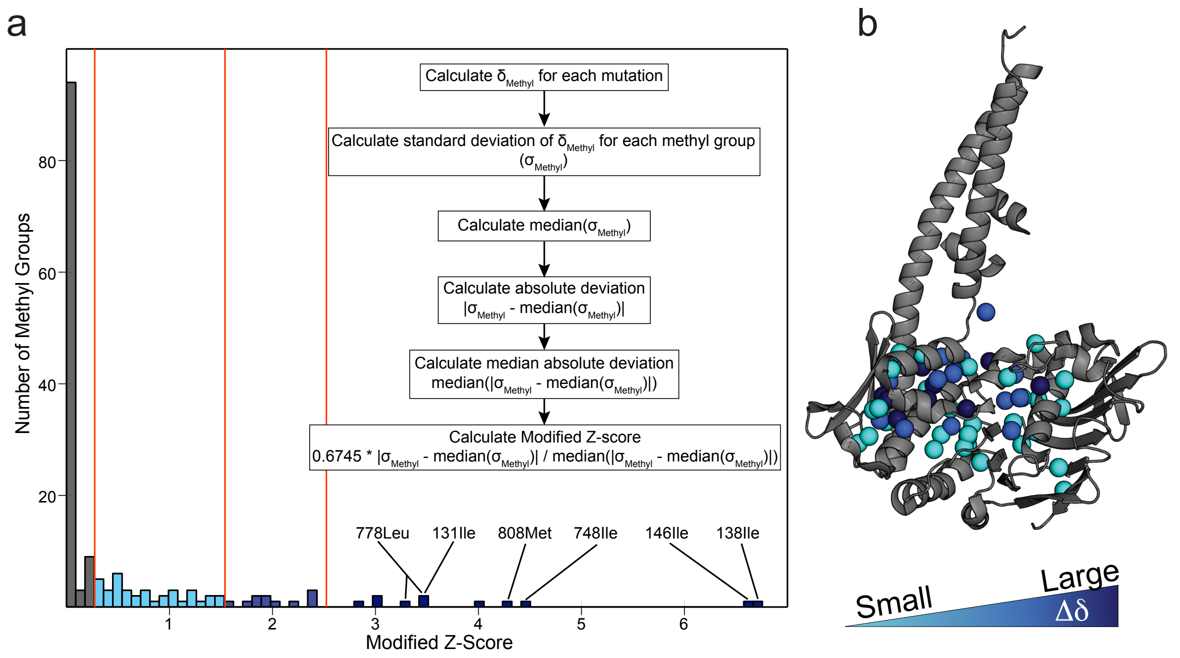

Supplementary Figure S3 | The median-absolute-deviation method used to identify the Rad50^{NBD} allosteric network. (**a**) Histogram of modified *Z-scores* illustrating the ability of the median-absolute-deviation method to identify outliers. Vertical lines represent the position of the cutoffs for modified *Z-scores* between 0.25 and 1.5, between 1.5 and 2.5, and greater than 2.5. The methyl groups that experience substantial chemical shifts are labeled. The flow-chart outlines the analysis. (**b**) Structure of Mre11^{HLH}-Rad50^{NBD} emphasizing methyl groups with significant CSPs as determined by the median-absolute-deviation approach: light, medium, and dark blue colored spheres represent methyl groups experiencing small-to-large CSP arising from mutation as defined by the vertical lines and bin colors in (**a**). Methyl groups not affected by the mutations are not shown.

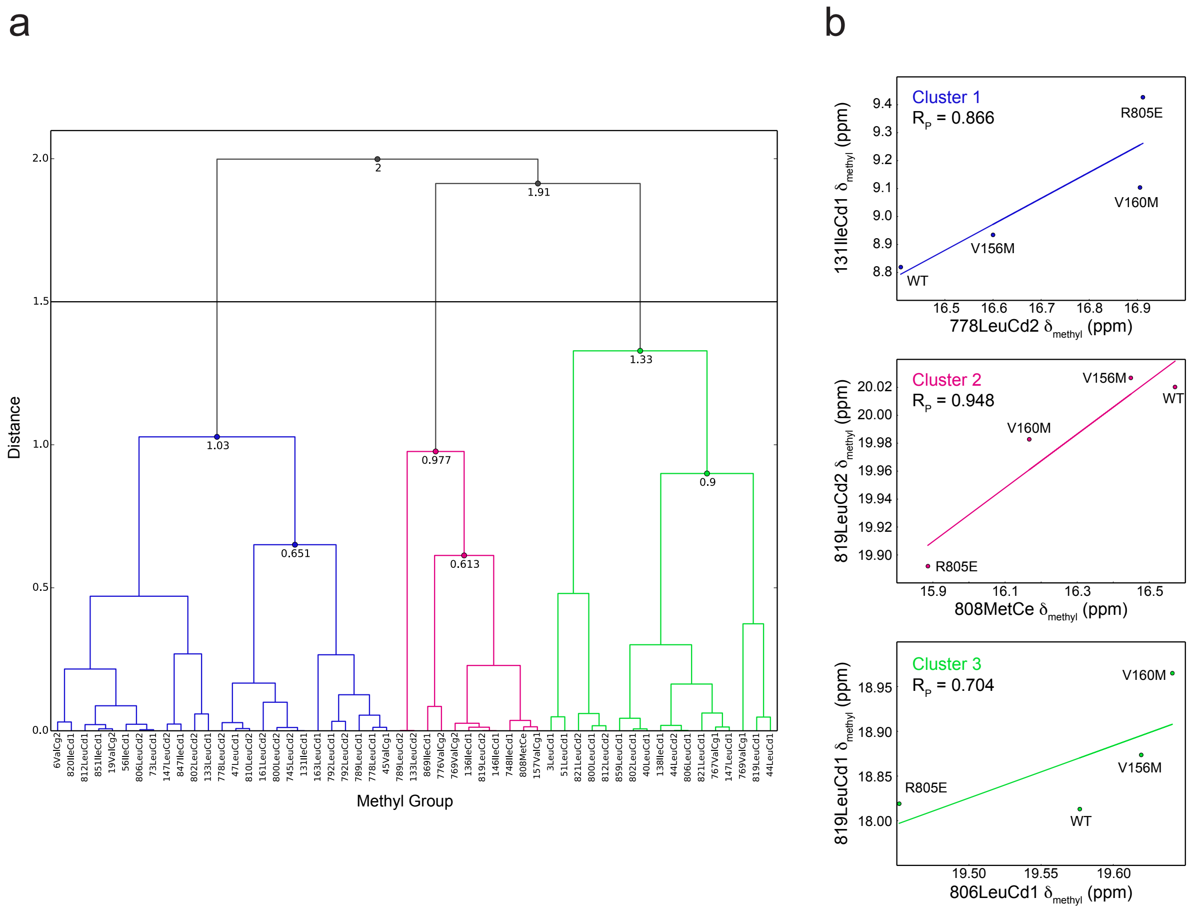

Supplementary Figure S4 | CHESCA of side chain methyl groups with significant CSPs reveals three distinct clusters. (**a**) Dendrogram of methyl groups determined by CHESCA. Only methyl groups that had a significant CSP (modified *Z-score* > 0.25) were included in CHESCA. A distance cutoff of 1.5 (horizontal black line) was used to define the clusters. Clusters 1, 2, and 3 are colored blue, magenta, and green, respectively. (**b**) Representative pairwise inter-residue correlation plots of the combined chemical shifts for each of the three clusters.

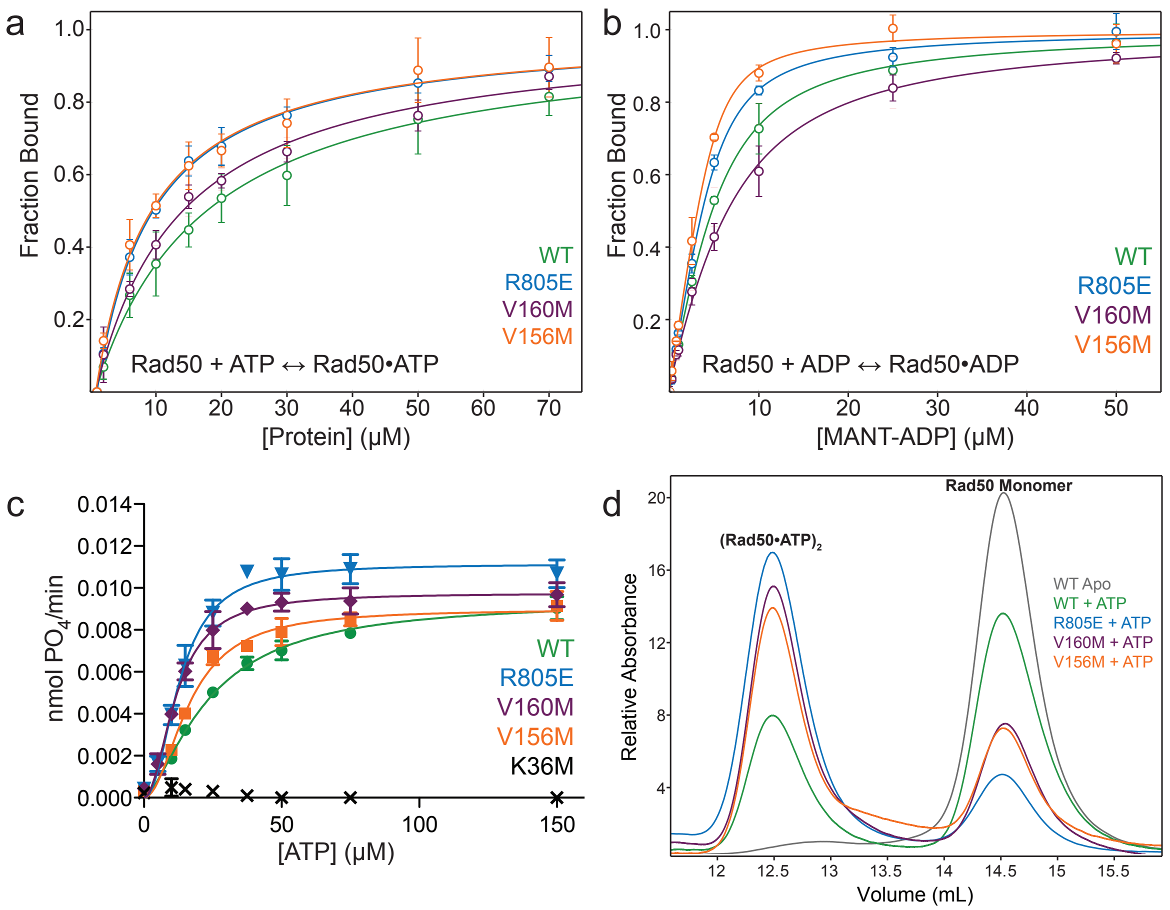

Supplementary Figure S5 | Rad50 and Mre11 activities are affected by hinge region and R805E mutations. (a) WT and mutant $Mre11^{HLH}$-$Rad50^{NBD}$ ATP binding affinities were determined from the change in polarization of a fluorescent nucleotide (FL-ATP) analog as a function of increasing protein concentration. Binding affinities were obtained by fitting fluorescence polarizations to equation (3). **(b)** WT and mutant $Mre11^{HLH}$-$Rad50^{NBD}$ ADP binding affinities were determined from the increase in FRET between a tryptophan residue in $Rad50^{NBD}$ and a fluorescent nucleotide (MANT-ADP) as a function of increasing MANT-ADP. After the proper corrections (see methods), ADP binding affinities were calculated from equation (3). Data in (**a** and **b**) were normalized based on the fitted F_{Max} and F_0 from equation (3) for plotting. **(c)** WT and mutant $Mre11^{HLH}$-$Rad50^{NBD}$ ATP hydrolysis activities were determined by monitoring the increase in product phosphate release via a colorimetric assay using BioMol reagent. After converting the observed change in color to nmol Pi min^{-1}, Michaelis-Menten kinetic parameters were obtained by fitting the data to equation (4). K36M is a Rad50 mutant known to disrupt

ATP hydrolysis[11]. Data points in (**a – c**) represent the average value of 3 – 5 experimental measurements across at least two enzyme preparations. Error bars are standard deviations of the replicate experimental measurements. (**d**) Size exclusion chromatograph of WT and mutant $Mre11^{HLH}$-$Rad50^{NBD}$ in the presence of 5 mM ATP and 5 mM $MgCl_2$. The grey curve shows the chromatograph for monomeric WT $Mre11^{HLH}$-$Rad50^{NBD}$ in the absence of ATP. The peak at ~12.5 mL is $Mre11^{HLH}$-$Rad50^{NBD}$ dimer induced by ATP binding, while the peak at ~14.5 mL is $Mre11^{HLH}$-$Rad50^{NBD}$ monomer.

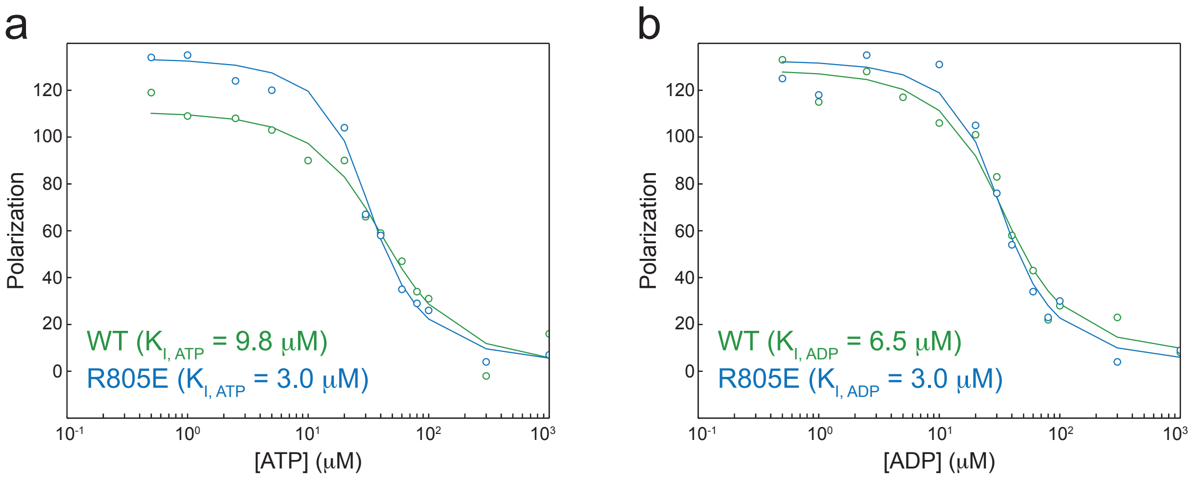

Supplementary Figure S6 | The fluorescent tag has negligible effects on Mre11^{HLH}-Rad50^{NBD} nucleotide binding affinities. WT and R805E Mre11^{HLH}-Rad50^{NBD} ATP binding affinities were determined from the change in polarization of a fluorescent nucleotide (BODIPY FL ATP) analog as a function of increasing concentration of unlabeled nucleotide. Data in **(a)** show ATP and **(b)** show ADP as the competing ligand. Binding affinities were obtained by fitting the decrease in fluorescence polarizations to a previously described equation for competitive binding[12].

a

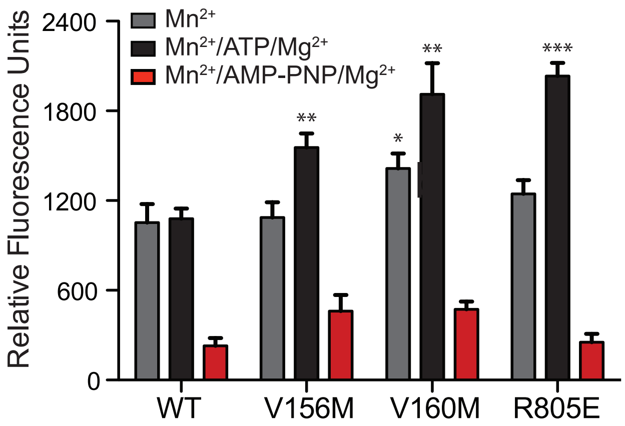

Supplementary Figure S7 | Mre11 exonuclease activity increases upon Rad50^{NBD} mutation. (**a**) Mre11 exonuclease activity for Mre11$_2$-Rad50$^{NBD}{}_2$ complexes of various Rad50^{NBD} mutants. Exonuclease activity was measured in the presence of 1 mM Mn^{2+} (grey bars), 1 mM Mn^{2+}/1 mM ATP/5 mM Mg^{2+} (black bars), or 1 mM Mn^{2+}/1 mM AMP-PNP/5 mM Mg^{2+} (red bars). Data points represent the average value of 3 – 5 measurements. Error bars are standard deviations of the replicate measurements.

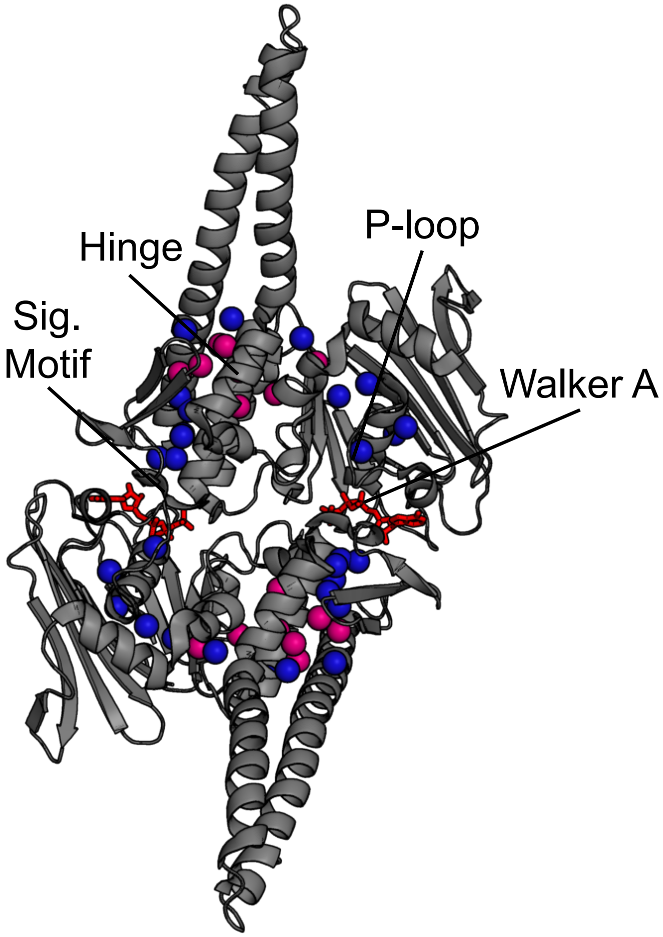

Supplementary Figure S8 | Methyl groups correlated to activity define a pathway connecting protomers in the dimer. Structure of dimeric Mre11^{HLH}-Rad50^{NBD} with bound AMP-PNP (pdb: 3qku)[6], which is shown in red sticks. Blue and magenta methyl groups represent cluster 1 and 2 residues, respectively, that have a correlation to ATP hydrolysis, dimerization and exonuclease activities ($|\text{mean}[R_{P,Hydrolysis}, R_{P,Dimerization}, R_{P,Exonuclease}]| > 0.65$). Methyl groups not affected by the mutations are not shown.

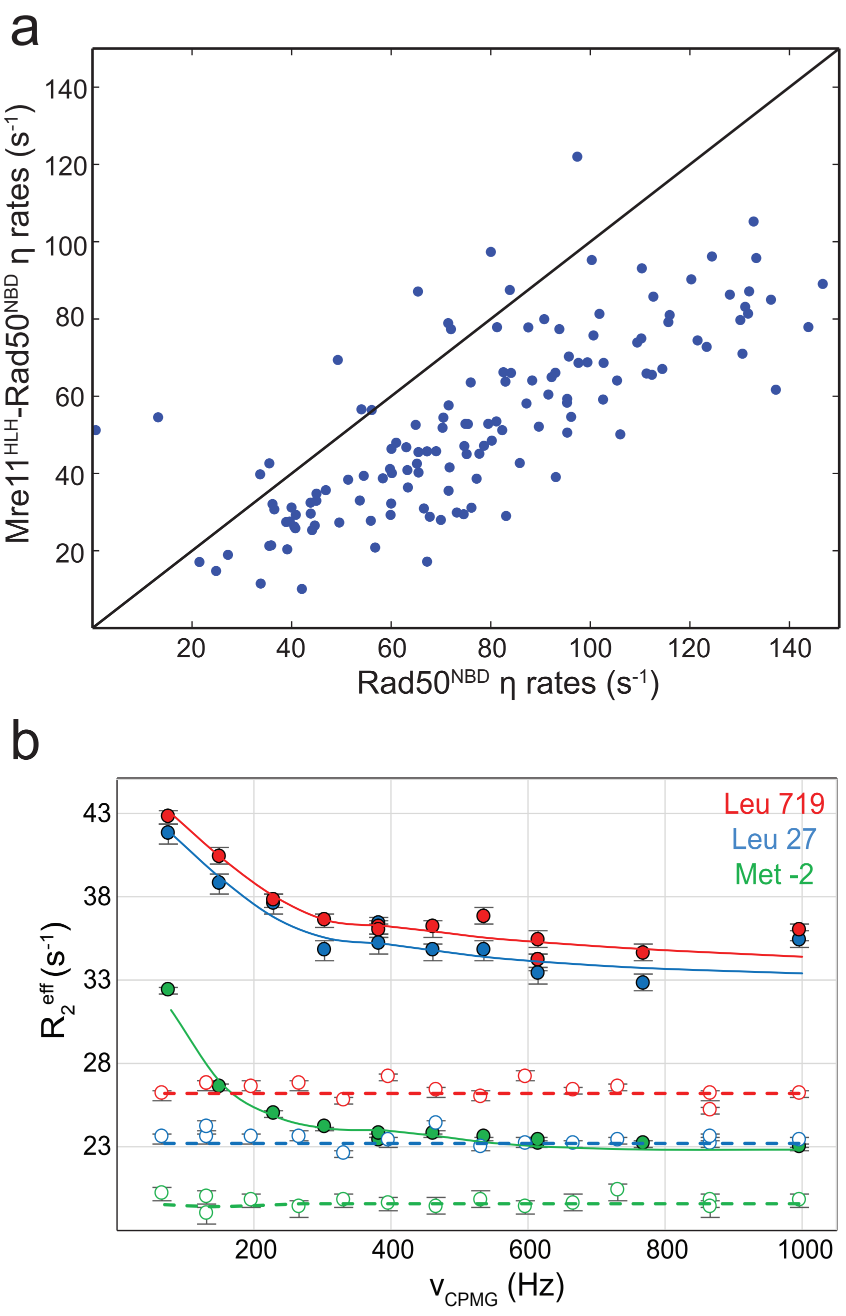

Supplementary Figure S9 | Mre11^{HLH} blocks a non-native, transient interaction between Rad50^{NBD} molecules. (**a**) Linear correlation plot of η rates from methyl group ^{1}H triple-quantum "forbidden" experiments[1] for the Mre11^{HLH}-Rad50^{NBD} complex *vs* Rad50^{NBD} without Mre11^{HLH}. The black line represents the y = x line. (**b**) Plot of methyl group single-quantum ^{13}C relaxation dispersion CPMG[5] R_2^{eff} rates *vs* applied CPMG field (ν_{CPMG}). Closed and open symbols are data

for Rad50^{NBD} without Mre11^{HLH} and the Mre11^{HLH}-Rad50^{NBD} complex, respectively. Solid lines are fits to a two-site exchange model, while the dashed lines are to guide the eye.
